# Open reading frame dominance indicates protein‐coding potential of RNAs

**DOI:** 10.15252/embr.202154321

**Published:** 2022-04-19

**Authors:** Yusuke Suenaga, Mamoru Kato, Momoko Nagai, Kazuma Nakatani, Hiroyuki Kogashi, Miho Kobatake, Takashi Makino

**Affiliations:** ^1^ Department of Molecular Carcinogenesis Chiba Cancer Centre Research Institute Chiba Japan; ^2^ Division of Bioinformatics National Cancer Centre Research Institute Tokyo Japan; ^3^ Department of Molecular Biology and Oncology Chiba University School of Medicine Chiba Japan; ^4^ Innovative Medicine CHIBA Doctoral WISE Program Chiba University School of Medicine Chiba Japan; ^5^ Laboratory of Evolutionary Genomics Graduate School of Life Sciences Tohoku University Sendai Japan

**Keywords:** gene birth, molecular evolution, noncoding RNA, ORF dominance, protein‐coding potential, Methods & Resources, RNA Biology

## Abstract

Recent studies have identified numerous RNAs with both coding and noncoding functions. However, the sequence characteristics that determine this bifunctionality remain largely unknown. In the present study, we develop and test the open reading frame (ORF) dominance score, which we define as the fraction of the longest ORF in the sum of all putative ORF lengths. This score correlates with translation efficiency in coding transcripts and with translation of noncoding RNAs. In bacteria and archaea, coding and noncoding transcripts have narrow distributions of high and low ORF dominance, respectively, whereas those of eukaryotes show relatively broader ORF dominance distributions, with considerable overlap between coding and noncoding transcripts. The extent of overlap positively and negatively correlates with the mutation rate of genomes and the effective population size of species, respectively. Tissue‐specific transcripts show higher ORF dominance than ubiquitously expressed transcripts, and the majority of tissue‐specific transcripts are expressed in mature testes. These data suggest that the decrease in population size and the emergence of testes in eukaryotic organisms allowed for the evolution of potentially bifunctional RNAs.

## Graphical abstract

The open reading frame dominance score correlates with translation efficiency of coding transcripts and with translation of noncoding RNAs. The score distributions in eukaryotes show overlap between coding and noncoding transcripts.

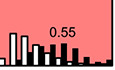

## Introduction

Recent advances in RNA‐sequencing technology have revealed that most of the eukaryotic genome is transcribed, primarily producing noncoding RNAs (Okazaki *et al*, 2002; Djebali *et al*, 2012; Ulitsky & Bartel, 2013; Kopp & Mendell, 2018). Noncoding RNAs longer than 200 nucleotides are long noncoding RNAs (lncRNAs) and are not translated into proteins (Ulitsky & Bartel, 2013; Kopp & Mendell, 2018). lncRNAs have been reported to participate in multiple biological phenomena, including the regulation of transcription, modulation of protein or RNA functions, and nuclear organization (Ulitsky & Bartel, 2013; Kopp & Mendell, 2018). However, paradoxically, a large fraction of lncRNAs is associated with ribosomes and translated into peptides (Frith *et al*, 2011; Ingolia *et al*, 2011; Bazzini *et al*, 2014; Ingolia, 2014; Ruiz‐Orera *et al*, 2014). Peptides translated from transcripts annotated as lncRNAs have multiple biological functions in several eukaryotes (Li & Liu, 2019; Huang *et al*, 2021), and some of these translations are specific to the cellular context (Douka *et al*, 2021). Conversely, known protein‐coding genes, such as *TP53*, can also function as RNAs (Candeias, 2011; Kloc *et al*, 2011; Huang *et al*, 2021). The discovery of these RNAs with binary functions has blurred the distinction between coding and noncoding RNAs, so the characteristics of RNA sequences that explain the continuum between noncoding and coding transcripts remain unclear.

During evolution, new genes originate from preexisting genes via gene duplication or from nongenic regions via the generation of new open reading frames (ORFs) (Ohno, 1970; Chen *et al*, 2013; Zhang & Long, 2014; McLysaght & Guerzoni, 2015; McLysaght & Hurst, 2016; Holland *et al*, 2017). The latter are *de novo* genes (Begun *et al*, 2006, 2007; Levine *et al*, 2006; Knowles & McLysaght, 2009; Toll‐Riera *et al*, 2009; Li *et al*, 2009, 2010a), which have been shown to regulate biological processes and diseases (Chen *et al*, 2013; Zhang & Long, 2014; McLysaght & Guerzoni, 2015), including brain function and carcinogenesis in humans (Li *et al*, 2010b; Suenaga *et al*, 2014). lncRNAs can serve as sources of *de novo* genes (Ruiz‐Orera *et al*, 2014), some of which evolve to encode proteins. In addition to ORFs exposed to natural selection, neutrally evolving ORFs are also translated from lncRNAs that stably express peptides (Ruiz‐Orera *et al*, 2018), providing a basis for the development of new functional peptides/proteins. High levels of lncRNA expression (Ruiz‐Orera *et al*, 2018), hexamer frequencies of ORFs (Sun *et al*, 2013; Wang *et al*, 2013; Ruiz‐Orera *et al*, 2014), and intrinsic disorder protein products (Heames *et al*, 2020) have been proposed as determinants of coding potential; however, the molecular mechanisms by which lncRNAs evolve into new coding transcripts remain unclear (Van Oss & Carvunis, 2019).

In the present study, we sought to identify a new indicator for determining RNA protein‐coding potential. First, we defined primary ORF as the longest of all ORFs of a given RNA and the indicator using the fraction of the primary ORF length constitutes the sum of all putative ORF lengths. We subsequently examined the associations between this indicator and protein‐coding potential. More than 3.4 million transcripts in 100 organisms belonging to all three domains of life were analyzed to investigate the relationship between this indicator and protein‐coding potential over evolutionary history.

## Results

### Coding transcripts show higher ORF dominance in humans and mice

**Figure 1:**
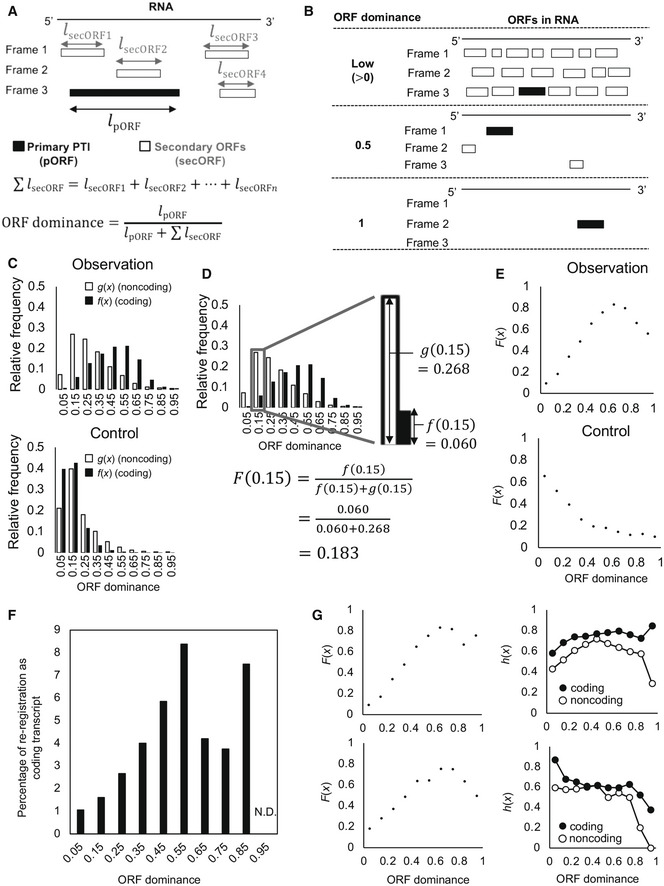
ORF dominance predicts the protein‐coding potential of human transcripts Conceptual schematic representation of ORFs in the three reading frames of an RNA and definition of ORF dominance. Black and white rectangles indicate primary and secondary ORFs, respectively. The primary ORF is the longest ORF, while secondary ORFs are all others; *l* is ORF length.Schematic representation of ORF distributions in RNAs with low (0‐0.5), medium (0.5), and high (1) ORF dominance.Relative frequencies of ORF dominance of coding, *f*(*x*), and noncoding, *g*(*x*), transcripts (upper) and of random controls (bottom).Explanation of *F*(*x*) for a ORF dominance of 0.15.ORF dominance correlations with protein‐coding potential, *F*(*x*), at ORF dominance ≤ 0.65 (upper) and those in random controls (lower).Relationship between ORF dominance and percentages of NR transcripts reregistered as NM during the past 3 years. N.D., not detected.Relationship between ORF dominance and *F*(*x*) in human transcripts syntenic to chimpanzee (upper left) and mouse (bottom left). The relative frequency of transcripts with negative selection, *h*(*x*), is plotted for each ORF dominance (upper and bottom right). The transcripts are syntenic to the genome of chimpanzee (upper right) and mouse (bottom right). The open circles indicate NR transcripts, and the full circles indicate NM transcripts.

We previously identified a *de novo* gene, *NCYM*, and revealed its biochemical function (Suenaga *et al*, 2014, 2020; Kaneko *et al*, 2015; Shoji *et al*, 2015; Matsuo *et al*, 2021). However, *NCYM* was previously registered as a noncoding RNA in the National Center for Biotechnology Information (NCBI) nucleotide database, and the coding potential assessment tool (CPAT), which is the established predictor for protein‐coding potential (Wang *et al*, 2013), showed *NCYM* had a coding probability of 0.022, labeling it as a noncoding RNA (Appendix Fig S1). Therefore, we sought to identify a new indicator for coding potential by comparing *NCYM* with a small subset of coding and noncoding RNAs to determine whether its sequence features would allow *NCYM* to be registered as a coding transcript. We found that predicted ORFs, other than major ORFs, were short in coding RNAs. In addition, it has been reported that upstream ORFs inhibit the translation of major ORFs (Calvo *et al*, 2009). Therefore, we hypothesized that the predicted ORFs may reduce the translation of major ORFs, thereby becoming short in the coding transcripts, including *NCYM*, during evolution. Major ORFs are often the longest ORFs (hereafter primary ORFs or pORFs) in coding transcripts. Thus, to investigate the importance of pORFs relative to other ORFs (hereafter secondary ORFs or secORFs) for the evolution of coding genes, we defined ORF dominance as the occupancy of the pORF length relative to the total ORF length (Fig 1A and B) and assumed that ORF dominance was high in coding transcripts. To examine this hypothesis, we first calculated ORF dominance for all human transcripts. We analyzed the human transcripts in the NCBI nucleotide database, including both coding and noncoding (RefSeq accession numbers starting with NM and NR, respectively) transcripts. The data were downloaded using the Table Browser (https://genome.ucsc.edu/cgi‐bin/hgTables) after setting the track tab as “RefSeq Genes”. A total of 50,052 coding (NM) and 13,550 noncoding (NR) RNAs were registered in the database in 2018 (Dataset EV1). To analyze putative lncRNAs with protein‐coding potential, we excluded small RNAs (shorter than 200 nucleotides) or RNAs with a short pORF (less than 20 amino acids) from the NR transcripts, as reported previously (Bazzini *et al*, 2014; Ruiz‐Orera *et al*, 2014; Schmitz *et al*, 2018), focusing on the remaining 12,827 transcripts.

We analyzed the relative frequencies of NM and NR transcripts, designated as *f*(*x*) and *g*(*x*), respectively (Fig 1C), where *x* indicates ORF dominance. In human transcripts, *g*(*x*) showed a distribution shifted to the left with an apex of 0.15; in contrast, the distribution of *f*(*x*) shifted to the right with an apex of 0.55 (Fig 1C, upper panel). We generated nucleic acid control sequences in which A/T/G/C bases were randomly assigned with equal probabilities. In these controls, the relative frequencies of ORF dominance shifted to the left in both coding and noncoding transcripts (Fig 1C, bottom panel). The controls that randomly shuffled the original sequence without affecting the number of A/T/C/G bases in each transcript also had relative frequencies of ORF dominance shifted to the left in both coding and noncoding transcripts (Appendix Fig S2A). Similar results were obtained using a dataset from the Ensembl database (Appendix Fig S2B). We also calculated the ORF dominance of mouse transcripts from RefSeq and Ensembl and found that the distribution of *f*(*x*) was shifted to the right with an apex of 0.55 (Appendix Fig S2C), similar to that of human transcripts.

### ORF dominance correlates with protein‐coding potential in human and mouse

Next, we examined the relationship between ORF dominance and protein‐coding potential. Based on the ORF dominance distributions of coding and noncoding transcripts, protein‐coding potential, *F*(*x*), was defined as the probability of a transcript being a coding RNA given an ORF dominance of *x*. A sample *F*(0.15) calculation for human transcripts is shown in Fig 1D. This result indicates that any given human RNA transcript with a calculated ORF dominance of 0.15 has a protein‐coding potential *F*(*x*) of 0.183. *F*(*x*) was correlated with ORF dominance ≤ 0.65 (Fig 1E and Appendix Fig S3A). The protein‐coding potentials of the sequences in the RefSeq database slightly decreased after peaking at 0.65 (Fig 1E), whereas those of sequences in the Ensembl database remained high (Appendix Fig S3A). The *F*(*x*) of the human transcripts was estimated using the following linear regressions:

For Ensembl data,

$$F\left(x\right)=1.301x+0.0072\left(x\leq 0.65\right),\;R^{2}=0.984;$$

For RefSeq data,

$$F\left(x\right)=1.313x+0.0189\left(x\leq 0.65\right),\;R^{2}=0.990.$$

The intercepts were near zero, and the slopes were approximately 1.3 for both equations. Using these equations, the *F*(*x*) of any given human transcript with an ORF dominance ≤ 0.65 can be calculated. For example, the *F*(*x*) of *NCYM* was estimated to be 0.746 or 0.765 based on Ensembl or RefSeq data, respectively (Appendix Fig S1D). In contrast, the *F*(*x*) of the control sequences was not correlated with ORF dominance (Fig 1E, bottom panel, and Appendix Fig S3A). Similar results were obtained for the mouse transcripts (Appendix Fig S3B). The *F*(*x*) of the mouse transcripts (ORF dominance ≤ 0.65) was estimated as follows:

For Ensembl data,

$$F\left(x\right)=1.142x+0.067,\;R^{2}=0.982$$

For RefSeq data,

$$F\left(x\right)=1.482x-0.061,\;R^{2}=0.990$$

For both human and mouse transcripts, ORF dominance correlated linearly with the protein‐coding potential at ORF dominance ≤ 0.65. Moreover, when the ORF dominance limit approached 0, the probability of the transcript being a coding RNA was 0 (Fig 1E and Appendix Fig S3).

### Characterization of high‐scoring human lncRNAs

Next, we investigated whether ORF dominance is useful for identifying coding RNAs among NR transcripts. From the 7,144 transcripts registered as noncoding genes in 2015, we excluded small RNAs (< 200 nucleotides) and those with short pORFs (< 20 amino acids). Among the remaining 6,617 NR genes, 219 were reassigned as NM over the past 3 years (Dataset EV2), including the previously identified *de novo* gene *MYCNOS*/*NCYM* (Suenaga *et al*, 2014). The percentage of reclassification increased for NR transcripts with high ORF dominance (Fig 1F). Thus, high ORF dominance is a useful indicator of coding transcripts. NR transcripts with high protein‐coding potential (0.6 ≤ ORF dominance < 0.8) were then extracted, and the domain structure of each pORF amino acid sequence was assessed using the basic local alignment search tool for protein sequences (BLASTP). A total of 217 transcripts showed putative domain structures in the pORF, whereas 310 did not (Dataset EV3). Transcripts with domain structures often derive from transcript variants, pseudogenes, or readthrough of coding genes; those without domain structures often derive from antisense or long intergenic noncoding RNAs (lincRNAs) (Table 1).

**Table 1:** Numbers of original transcripts that produced NR transcripts with high coding frequency (0.6 ≤ ORF dominance < 0.8).

Transcript	Domain		Total	*P*‐value
	With	Without		
Antisense	4	61	65	7.79E‐08
lincRNA	3	65	68	7.60E‐09
Pseudogene	50	17	67	4.32E‐07
Readthrough	7	0	7	6.00E‐03
Transcript variant of coding gene	146	35	181	1.05E‐19
Divergent	0	2	2	N.S.
Intronic	0	6	6	N.S.
Small nuclear RNA	0	3	3	N.S.
miRNA host gene	0	3	3	N.S.
Other lncRNA	7	118	125	1.12E‐13
Total	217	310	527	

*P*‐values were calculated using Yate’s continuity correction. N.S., not significant.

We next examined the functions of the genes originating NR transcripts with high coding potential (0.6 ≤ ORF dominance < 0.8). We divided the NR transcripts into those with and without putative domains to investigate novel coding gene candidates, originating either from preexisting genes or from nongenic regions. Analysis using the Database for Annotation, Visualization, and Integrated Discovery (DAVID) functional annotation tool (Huang *et al*, 2009a, 2009b) showed that NR transcripts without domain structures were derived from genes related to transcriptional regulation, multicellular organismal processes, and developmental processes (Dataset EV4). Among the target genes of transcription factors, *NMYC* (*MYCN*), *TGIF*, and *ZIC2* were ranked in the top three, and are all necessary for forebrain development (Dataset EV4) (Brown *et al*, 1998; Gripp *et al*, 2000; van Bokhoven *et al*, 2005). NR transcripts with domain structures originating from genes with alternative splicing were related to organelle function and are expressed in multiple cancers, including respiratory tract tumors, gastrointestinal tumors, retinoblastomas, and medulloblastomas (Dataset EV5). Similar analyses were conducted for mouse (Datasets [Link], [Link], [Link], [Link]) and *Caenorhabditis elegans* (Datasets [Link], [Link], [Link], [Link]). In mouse, original genes related to protein dimerization activity (Dataset EV7) and nucleotide binding or organelle function (Dataset EV8) were enriched and showed high ORF dominance lncRNAs with and without conserved domains, respectively. In *C. elegans*, original genes related to embryo development (Dataset EV10) and chromosome V or single‐organism cellular processes (Dataset EV11) were enriched. Therefore, the relationship between brain development and cancer in the function of lncRNAs with high ORF dominance seems to be specific to humans.

### ORF dominance affects the protein‐coding potential predicted by *K*a/*K*s

To examine the relationship between ORF dominance and natural selection in the prediction of protein‐coding potential, we calculated the ratio of nonsynonymous (*K*a) to synonymous (*K*s) mutations by comparing human transcripts with syntenic–genomic regions of chimpanzee and mouse (Fig 1G). Transcripts were selected based on syntenically conserved regions: 44,593 (vs. chimpanzee) and 14,016 (vs. mouse). We found a linear relationship between *F*(*x*) and ORF dominance in the conserved transcripts (Fig 1G, left panels). As predicted, coding transcripts exhibited *K*a/*K*s < 0.5 at a higher frequency than noncoding transcripts, with large differences observed for ORF dominance > 0.9 or < 0.1 and the smallest difference for ORF dominance between 0.35 and 0.45, approximately (Fig 1G, right panels). These results indicated that for transcripts with ORF dominance near the highest or lowest values, the conservation of pORF sequences (negative selection, *K*a/*K*s < 0.5) determines the coding potential. Therefore, noncoding transcripts showing both negative selection (*K*a/*K*s < 0.5) and the highest ORF dominance may correspond to new coding transcript candidates. We list 23 such transcripts in Dataset EV12, including four transcript variants of a previously identified lncRNA that encodes a tumor‐suppressive small peptide, HOXB‐AS3 (Huang *et al*, 2017).

### Translation of small peptides shifts ORF dominance distributions

**Figure 2:**
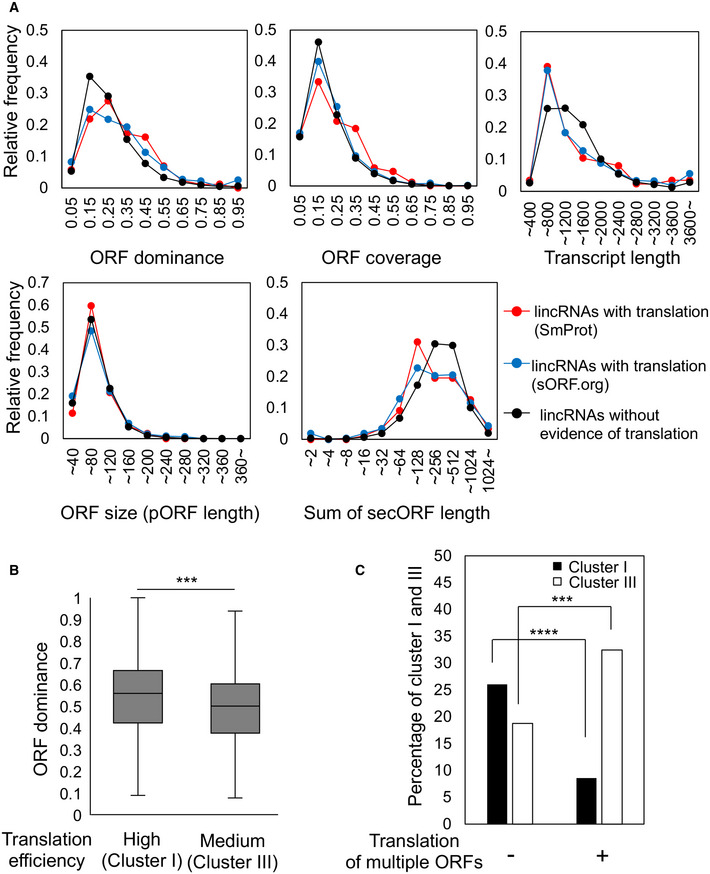
Effects of translation on the distributions of ORF dominance The ORF dominance distribution for lincRNAs with translation registered in the SmProt database (http://bioinfo.ibp.ac.cn/SmProt/) (red line, *n* = 87) or sORF database (http://www.sorfs.org/) (blue line, *n* = 594) shifted to higher scores relative to lincRNAs without evidence of translation (black line, *n* = 11,657, not registered in these databases) (top left). The relative frequency of corresponding ORF coverage (top center), transcript length (top right), ORF size (bottom left), and sum of secORF length (bottom right) are also shown.Cluster I genes (*n* = 1,149) show higher ORF dominance than cluster III genes (*n* = 2,918).Central bands, whiskers, and boxes are median values, ranges, and interquartile ranges, respectively. *P*‐values were calculated by the Mann–Whitney U‐test. ***P < 10^−9^.Genes with translation of multiple ORFs (*n* = 7,961) show lower or higher percentage of cluster I or cluster III genes, respectively, than genes without evidence of translation of multiple ORFs (*n* = 1,786, not registered in sORF databases). *P*‐values were calculated using Yate’s continuity correction. ****P = 1.46E‐68 and ***P = 4.86E‐18.

To investigate the effect of translation on ORF dominance, we calculated the ORF dominance of lincRNAs with translation registered in two independent databases (SmProt and sORFs.org) and compared them with that of lincRNAs without evidence of translation. Results showed that lincRNAs with translation had higher ORF dominance than those without translation evidence (Fig 2A, top left panel).

Transcript length and the coverage, and size (pORF length) of ORFs have been used as indicators to predict the coding potential of transcripts (Wang *et al*, 2013; Zeng & Hamada, 2018), including *de novo* genes (Schmitz *et al*, 2018). We calculated these three values for lincRNAs with translation products, and their distributions were compared with those of lincRNAs without evidence of translation. The comparison revealed a slight shift in the high values of ORF coverage in the lincRNAs registered in SmProt, whereas negligible changes were found in the distribution of lincRNAs registered in sORF.org (Fig 2A, top center panel). In addition, there was no shift in ORF size (Fig 2A, bottom left panel), and transcripts were rather short in lincRNAs with translation (Fig 2A, top right panel), reducing the sum of secORFs length (Fig 2A, bottom right panel). Therefore, the translated lincRNAs showed high ORF dominance, to which contributed their shorter transcript lengths by reducing the sum of secORFs.

Next, we examined whether ORF dominance was associated with translation efficiency in coding RNAs. Transcript translation in spermatocytes and spermatids is strongly downregulated on average. However, Wang *et al* (2020) identified gene sets (cluster I genes) efficiently translated in the spermatocytes and spermatids of mouse that therefore escaped the overall translational repression (Wang *et al*, 2020). We found that cluster I genes had higher ORF dominance than cluster III genes showing translational repression in spermatocytes and spermatids (Fig 2B). Furthermore, coding transcripts with translation from multiple ORFs showed significantly low or high percentages of cluster I or cluster III genes, respectively, compared with those without evidence of translation (Fig 2C). These results supported the hypothesis that ORF dominance is associated with translation efficiency in coding transcripts.

### Relationship between ORF dominance and relative frequencies of coding/noncoding transcripts in 100 organisms

**Figure 3:**
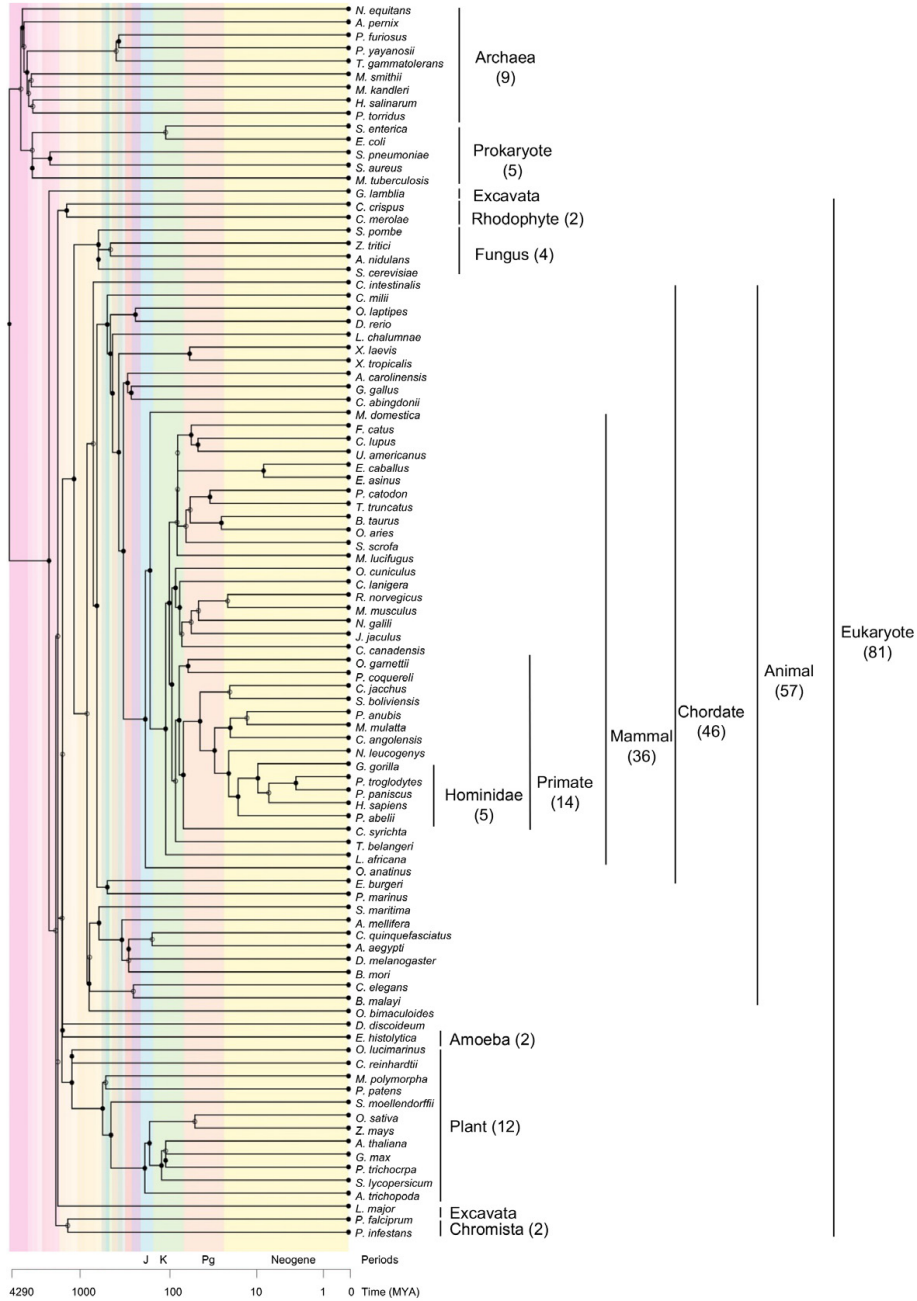
Phylogenetic tree Numbers of species are indicated in each lineage. The lineages of five species, including one archaea (*Nitrososphaera viennensis* EN76), two fungi (*Puccinia graminis* f. sp. *Tritici* and *Pyricularia oryzae*), and two animals (*Strongylocentrotus purpuratus* and *Lingula anatine*) are unknown and therefore were excluded from the figure.

**Figure 4:**
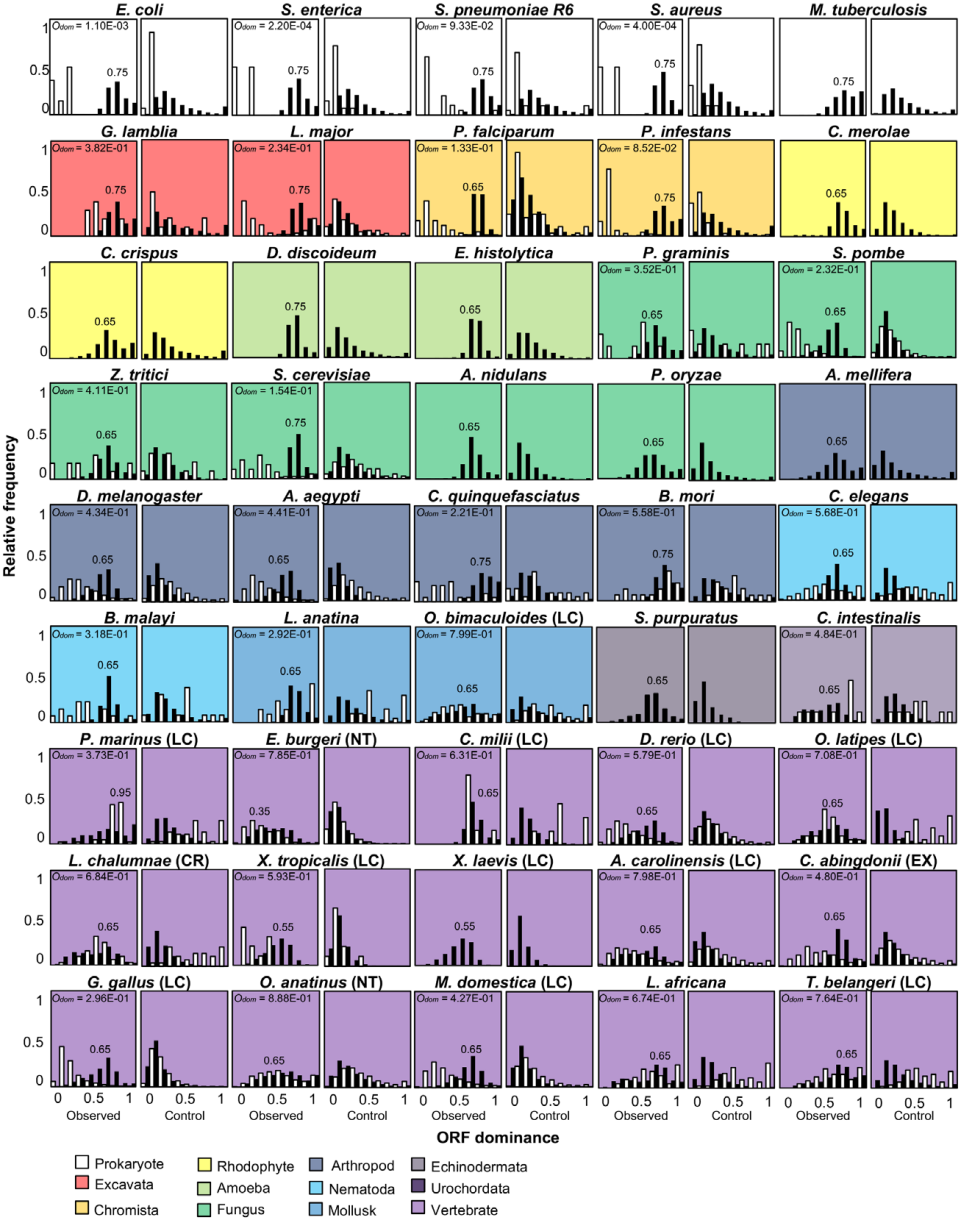
Relationships between ORF dominance and the relative frequencies of coding and noncoding transcripts from bacteria to mammals Histograms of *f*(*x*) (white) or *g*(*x*) (black) in observed data (left) and in nucleic acid‐scrambled controls (right) for each species analyzed. ORF dominance with the highest *f*(*x*) is presented in the histograms. *O*_dom_ was calculated using the ORF dominance distribution from observed data, and it is indicated in the left panels. LC, Least Concern; NT, Near Threatened; CR, Critically Endangered; and EX, Extinct in International Union for Conservation of Nature (IUCN) Red List.

**Figure 5:**
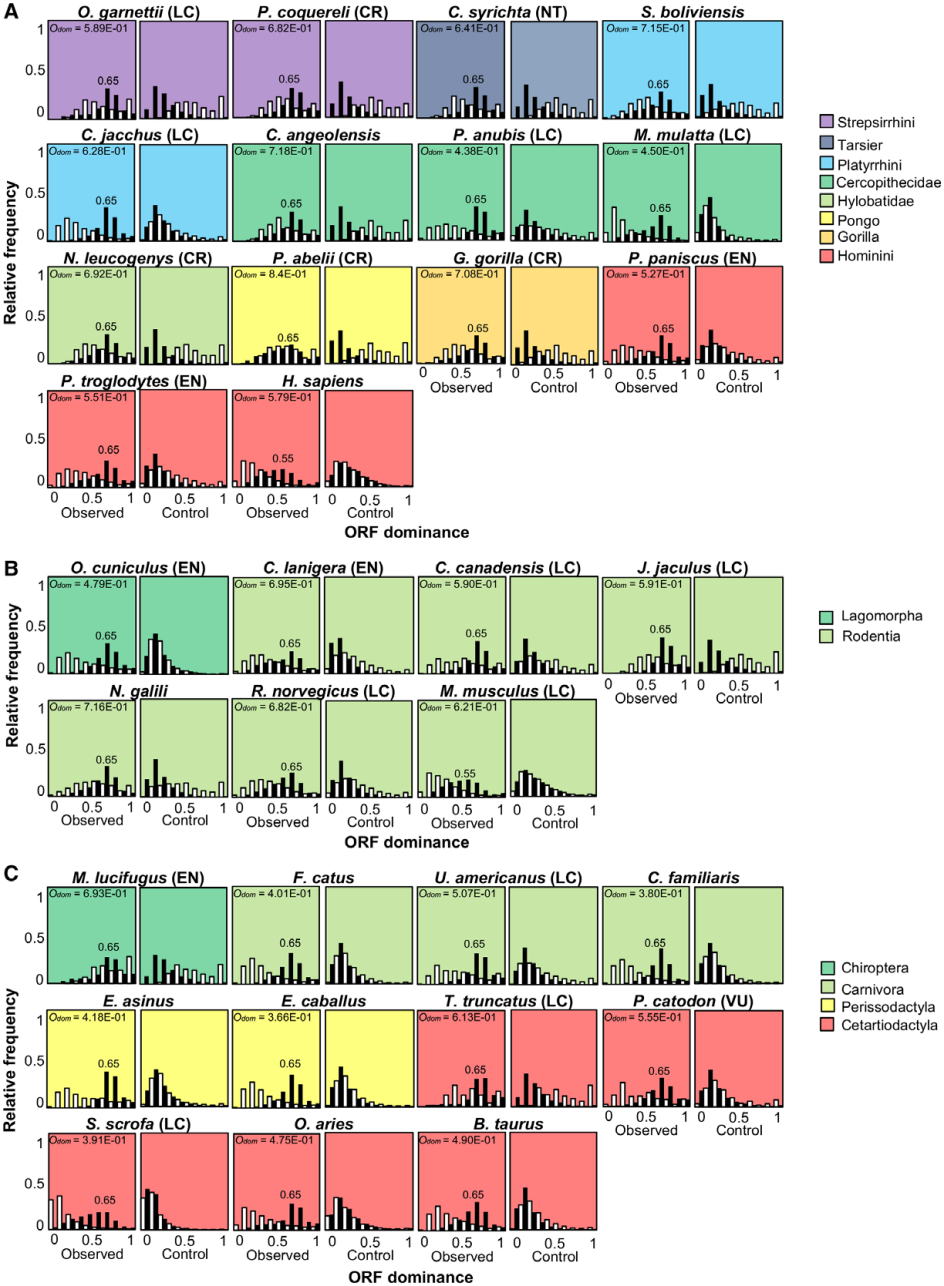
Relationships between ORF dominance and the relative frequencies of coding and noncoding transcripts A–CPrimates (A), Glires (B), and Laurasiatheria (C). LC, Least Concern; NT, Near Threatened; VU, Vulnerable; EN, Endangered; and CR, Critically Endangered in IUCN Red List.

To analyze the relationship between ORF dominance and protein‐coding potential in a broad lineage of 100 organisms, we selected five bacteria, ten archaea, and 85 eukaryote species (Dataset EV1) and calculated ORF dominance for more than 3.4 million transcripts (Dataset EV1). Phylogenetic trees of the cellular organisms are presented using a logarithmic timescale and display the number of species in each lineage (Fig 3). To examine the evolutionary conservation of the linear relationship between ORF dominance and protein‐coding potential in human being and mouse, we selected a relatively large number of mammalian species (36). Species with fewer than three lncRNAs were not used to calculate *g*(*x*) and were not included in the histograms illustrating the relationship between *g*(*x*) and ORF dominance (Figs 4 and 5). For all organisms, the relative frequency of coding transcripts, *f*(*x*), was shifted to the right (higher ORF dominance) compared with random or random shuffling controls (Figs 4 and 5A–C; Appendix Figs S4 and S5).

In bacteria and archaea, *f*(*x*) and *g*(*x*) exclusively exhibited high and low ORF dominance, respectively, indicating a clear boundary between coding transcripts and lncRNAs in terms of ORF dominance (Fig 4 and Appendix Fig S4). In addition, the highest *f*(*x*), which corresponded to high ORF dominance, was 0.75 in all examined bacteria (Fig 4) and ≥ 0.75 in archaea (Appendix Fig S4). Among eukaryotes, unicellular organisms and nonvertebrates showed the highest frequencies of coding transcripts at 0.65 or 0.75 (Fig 4), while for most vertebrates, the highest values were ≤ 0.65 (Figs 4 and 5). In addition, the *f*(*x*) distribution in vertebrates was broad and shifted to the left (low ORF dominance) relative to those of bacteria and archaea (Figs 4 and 5). In sharp contrast to *f*(*x*), the relative frequency of lncRNAs, *g*(*x*), was shifted to the right (high ORF dominance) in eukaryotes, including *Giardia lamblia*, which belongs to the earliest diverging eukaryotic lineage and lacks mitochondria (Fig 4). As the distribution of *f*(*x*) in the Excavata, including *G. lamblia*, showed a similar pattern to that of bacteria, the right shift of *g*(*x*) seems to have occurred earlier than the left shift of *f*(*x*) in the evolution of eukaryotes. Collectively, the left and right shifts of *f*(*x*) and *g*(*x*), respectively, seem to have contributed to blur the boundary between coding and noncoding transcripts in eukaryotes.

### The distribution overlap of ORF dominance is inversely correlated with effective population size

**Figure 6:**
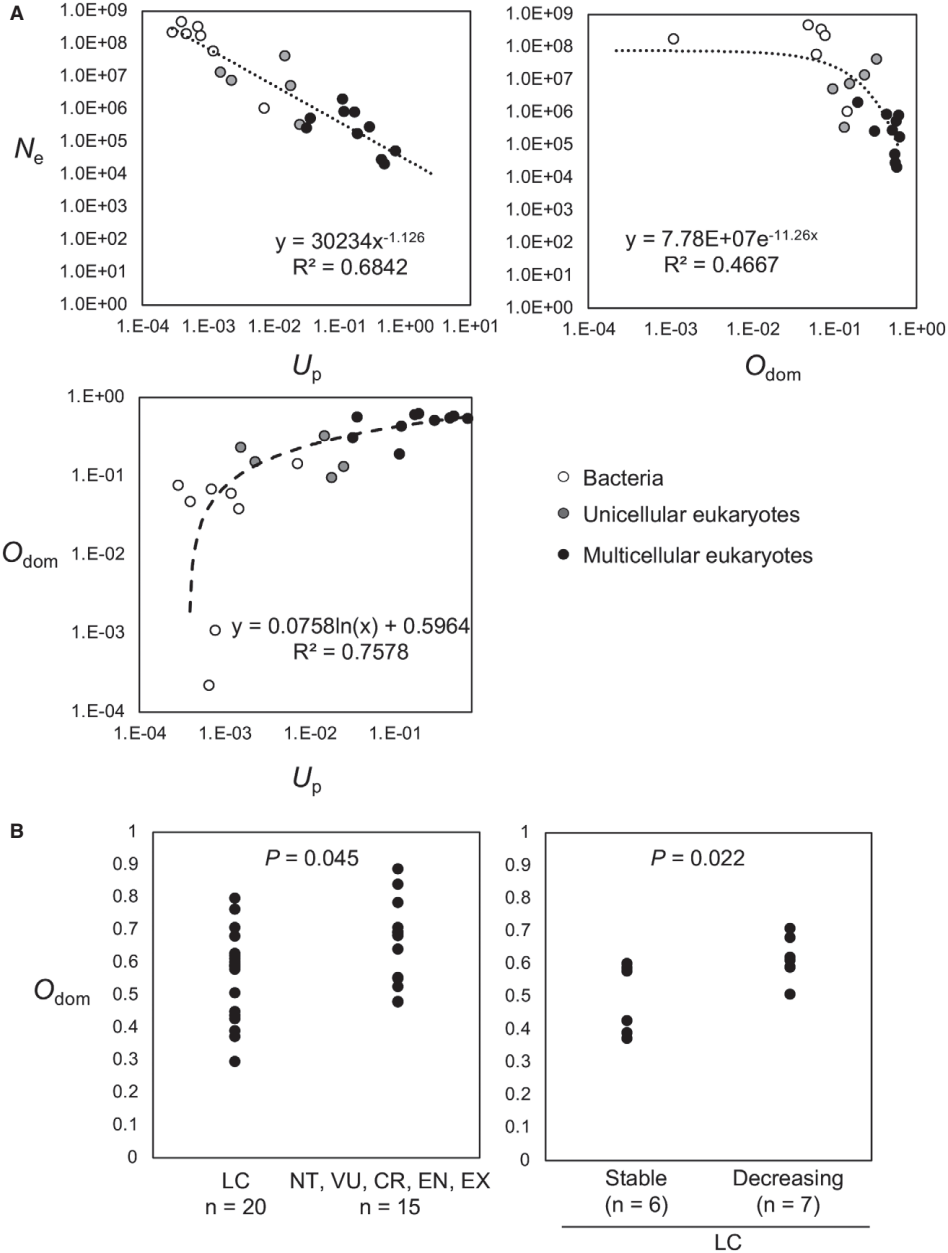
The overlap of ORF dominance distribution is negatively correlated with effective population size Inversely proportional relationship between genome‐wide mutation rates in protein‐coding DNA per generation (*U*_p_) and effective population size (*N*_e_) in 24 species (left upper). Values are from Lynch *et al* (2016). *O*_dom_ positively and negatively correlates with *U*_p_ (left bottom panel) and *N*_e_ (right upper panel); these relationships are approximately logarithmic and exponential, respectively. White, gray, and black dots indicate bacteria, unicellular eukaryotes, and multicellular eukaryotes, respectively.
*O*_dom_ is increased in vertebrates at risk of extinction (left) and with decreasing population trends (right). LC, Least Concern (*n* = 20); NT, Near Threatened (*n* = 3); VU, Vulnerable (*n* = 1); EN, Endangered (*n* = 5); CR, Critically Endangered (*n* = 5); and EX, Extinct (*n* = 1). *P*‐values were calculated by the Mann–Whitney U‐test.

In general, eukaryotes (particularly multicellular organisms) have smaller effective population sizes than prokaryotes, with higher mutation rates due to the effect of genetic drift (Lynch *et al*, 2016). We defined an indicator of coding/noncoding boundary ambiguity (the overlapping score, *O*_dom_) and examined the relationship between *O*_dom_ and the effective population size and mutation rate using data from a previous study (Lynch *et al*, 2016). An overlapping score based on ORF coverage, *O*_cov_, was also defined for comparison (Appendix Fig S6). Of the 35 species used in the previous study, 11 had no more than five lncRNAs with pORFs longer than 20 amino acids, and thus, the transcripts of the remaining 24 species (Dataset EV13 and Appendix Fig S6) were used for further analysis. Similar to a previous report (Lynch *et al*, 2016), the effective population size was inversely proportional to the mutation rate of genomic DNA in the 24 species selected (exponent = −1.126, *R* ^2^ = 0.6842; Fig 6A). *O*_dom_ was positively and negatively correlated with mutation rate and effective population size, with relationships that could be approximately logarithmic (*R* ^2^ = 0.7578) and exponential (*R*² = 0.4667), respectively. In contrast, *O*_cov_ showed a weak correlation with both mutation rate and effective population size (Appendix Fig S7).

Substituting the maximum value of *O*_dom_ (1) into the exponential function (Fig 6A, right upper panel) yielded the minimum effective population size, i.e., 1,001.28. This value was consistent with the minimum effective population size observed in conservation biology, which is approximately 1,000 (Frankham *et al*, 2014). This finding led us to consider the possibility that *O*_dom_ may be elevated in endangered organisms. Therefore, we calculated *O*_dom_ for 35 of the vertebrate species on the International Union for Conservation of Nature Red List (Fig 6B, left panel; Dataset EV1) and found that species at risk of extinction had significantly higher *O*_dom_ than species with lower risk of extinction (Least Concern, LC). In addition, among LC species, *O*_dom_ was higher for species with decreasing numbers than those with stable populations (Fig 6B, right panel; Dataset EV1).

### Relationship between ORF dominance and protein‐coding potential

**Figure 7:**
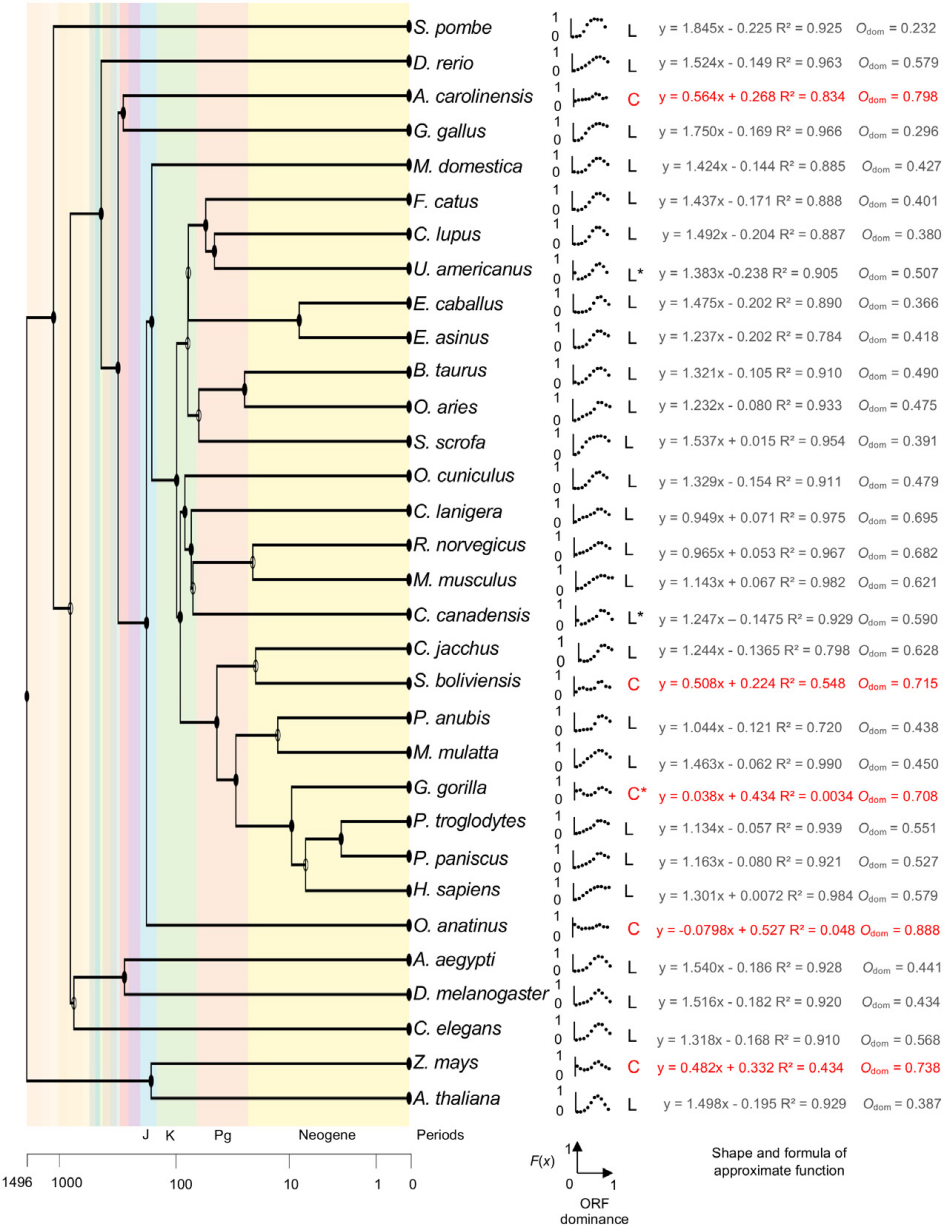
Relationship between ORF dominance and protein‐coding potential, *F*(*x*), for 32 eukaryotes The phylogenetic tree includes the 32 species (left), dot plots, and the shape and formulas of approximate functions. L and C indicate linear (in black) and constant (in red) functions. Fewer than five lncRNAs had a ORF dominance of 0.05 in *U. americanus*, *C. canadensis*, and *G. gorilla*; therefore, we eliminated the *F*(0.05) for these species for linear function approximations (asterisks). *O*_dom_ was calculated using the ORF dominance distributions of observed data.

The overlapping of relative frequencies in *f*(*x*) and *g*(*x*) led us to examine the relationship between ORF dominance and protein‐coding potential, *F*(*x*), in eukaryotes. To avoid biases due to the small sample size, we selected 32 species with more than 1,000 lncRNAs containing pORFs to calculate *F*(*x*) (Fig 7 and Appendix Fig S8). In human being and mouse, the relationship between ORF dominance and *F*(*x*) was approximately linear and passing through the origin of ORF dominance ≤ 0.65. Therefore, we used a linear function to estimate *F*(*x*) for the 32 species and found that it was a good fit for 27 of the 32 species (indicated as a linear group, L, in Fig 7 and Appendix Fig S8). In *Ursus americanus*, *Cornus canadensis*, and *Gorilla gorilla*, fewer than five lncRNAs exhibited ORF dominance of 0.05; thus, we eliminated the *F*(0.05) in these species for the estimation of *F*(*x*) using the linear function (indicated with asterisks in Fig 7). The *F*(*x*) of the remaining five species, which showed *O*_dom_ > 0.7, did not fit the linear function (indicated as a constant group, C, in Fig 7), and it was characterized by low slope values. These five species belonged to plants (*Zea mays*), reptiles (*Anolis carolinensis*), and mammals (*Ornithorhynchus anatinu*, *Saimiri boliviensis*, and *G. gorilla*) (Fig 7). In these species, ORF dominance showed a weaker association with the protein‐coding potential. In addition, these species may have small effective population sizes due to the risk of extinction (*O. anatinu* and *G. gorilla*) or due to artificial selection as pets (*A. carolinensis* and *S. boliviensis*) or as crops (*Z. mays*).

### Characteristics of RNA viral genomes in human and bacterial cells

**Figure 8:**
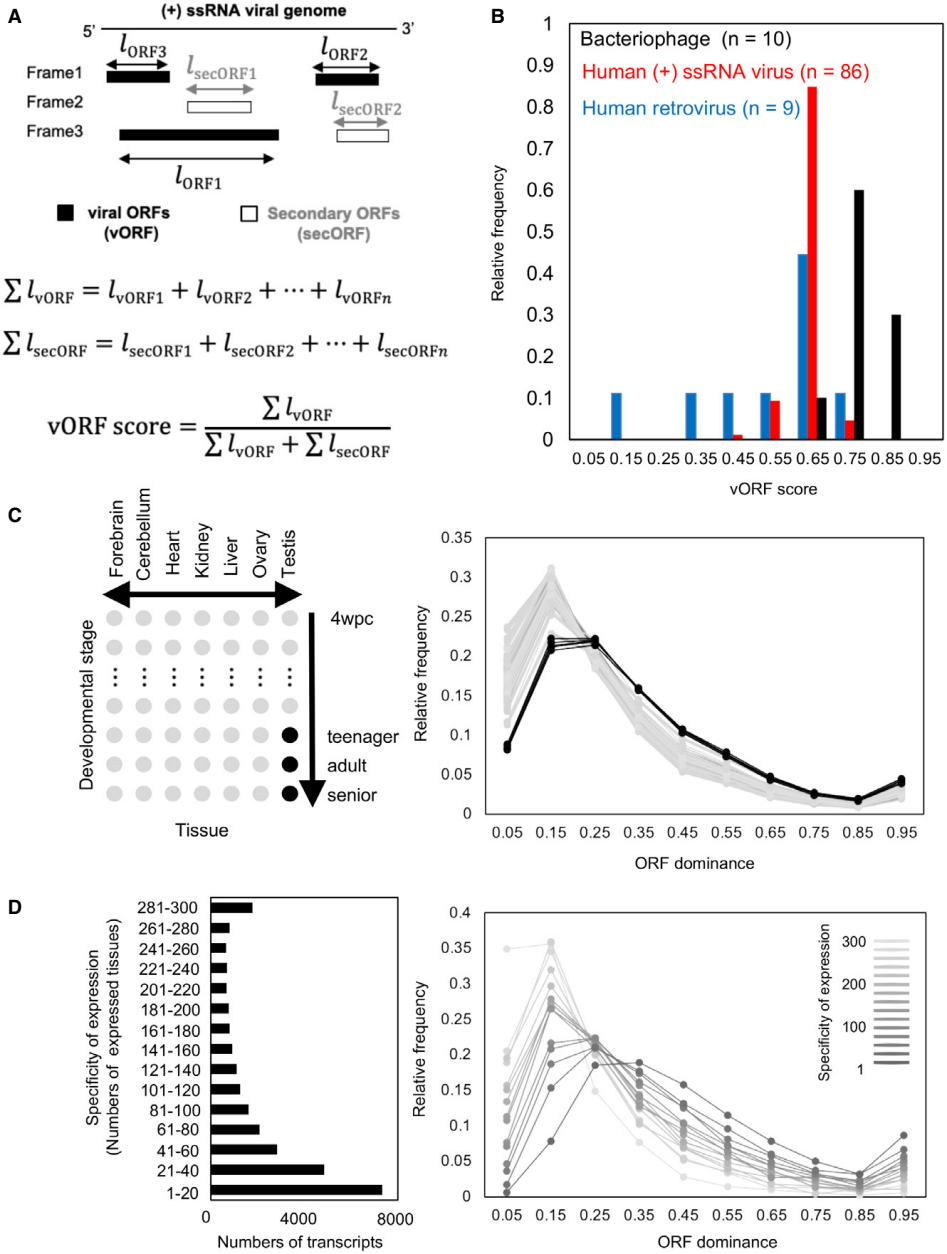
Molecular mechanisms affecting ORF dominance distributions Schematic explanation of secORF length and bona fide viral ORFs in a (+) ssRNA viral genome and the definition of viral ORF (vORF) score. Black and white rectangles indicate vORFs and secORFs, respectively. *l* is the length of the ORFs.Histograms of relative frequencies of human (+) ssRNA viruses (red) and bacteriophages (black).ORF dominance distributions of lncRNAs in human tissues. Distributions in mature testes and other tissues are indicated as black and gray lines, respectively.The relationship between tissue specificity and ORF dominance distribution in humans. Line intensity increases with increasing specificity of gene expression.

In sharp contrast to the coding transcripts of bacteria and archaea, the ORF dominance of coding transcripts in eukaryotes overlapped with that of noncoding RNAs due to the broad distribution of low ORF dominance. To investigate the molecular mechanism underlying the distinct distribution of coding transcripts between bacteria and eukaryotes, we analyzed the genome sequences of RNA viruses that infect human or bacterial cells. Positive‐sense single‐stranded RNAs, (+) ssRNAs, are parts of the viral genome that generate mRNAs and are translated into viral proteins via the host translation system. Therefore, efficient translation in host cells contributes to the replication of (+) ssRNA viruses. We hypothesized that ORFs other than bona fide ORFs affect the coding potential of the viral genome in host cells. Multiple bona fide ORFs are present in viral genomes. Thus, we extended the concept of ORF dominance to the multiple ORFs in viral RNA genomes (Fig 8A) and set the viral ORF (vORF) score.

Among the (+) ssRNA viruses registered in the NCBI database, 198 were human viruses and 13 were bacteriophages. We eliminated the viruses that produced viral proteins by exceptional translation mechanisms such as ribosome frameshifting, alternative initiation sites, ribosome slippage, and RNA editing, focusing on the remaining 95 human viruses, including nine retroviruses (Dataset EV14) and ten bacteriophages (Dataset EV15). The relative frequencies of human viruses and bacteriophages showed distinct peaks at the vORF scores of 0.65 and 0.75, respectively (Fig 8B). These values correspond to the ORF dominance associated with the highest protein‐coding potential in humans (Fig 1E and Appendix Fig S3A) and the highest frequency of coding transcripts in bacteria (Fig 4). In addition, the relative frequency of human viruses showed a broader distribution of low ORF dominance compared with bacteriophages, particularly in human retroviruses (Fig 8B). Therefore, RNA viral genomes appear to have sequence characteristics that maximize their protein‐coding potential in host cells.

### Relationship between ORF dominance and tissue‐specific expression

The shift to the right observed in the distribution of ORF dominance in noncoding RNAs is pronounced in eukaryotes, especially in multicellular organisms (Figs 4 and 5). To examine the possibility that different tissues of multicellular organisms show different ORF dominance distributions for noncoding RNAs, we analyzed transcriptome data to calculate the ORF dominance of human noncoding transcripts expressed in multiple tissues (Fig 8C). ORF dominance distributions were similar for almost all tissues, except for mature testes where distribution was shifted to higher values (Fig 8C). Similar results were obtained for opossum, rat, mouse, and macaque, although shifts in the ORF dominance distribution were weaker in these species than in humans (Appendix Fig S9). Furthermore, the noncoding transcripts that were expressed in a tissue‐specific manner had higher ORF dominance than ubiquitously expressed noncoding transcripts in humans (Fig 8D) and in opossum, rat, mouse, and macaque (Appendix Fig S10). The relationship between the specificity of expression and ORF dominance was also found for human coding transcripts (Appendix Fig S11). These results suggested that the evolution of tissue‐/cell type‐specific expression in multicellular eukaryotes contributed to increased ORF dominance for noncoding transcripts. Since the majority of tissue‐specific transcripts were expressed in matured testes (7,573 of 8,523 transcripts (89%) in the highest specificity group for humans), the evolution of testicular tissues also seems to have contributed to the existence of high ORF dominance noncoding RNAs, thus contributing to the appearance of *de novo* coding genes.

## Discussion

Here, we showed that ORF dominance is associated with protein‐coding potential in cellular organisms. In bacteria and archaea, the distributions of ORF dominance for noncoding and coding transcripts were distinct (low and high scores), whereas they were merged in eukaryotes.

**Figure 9:**
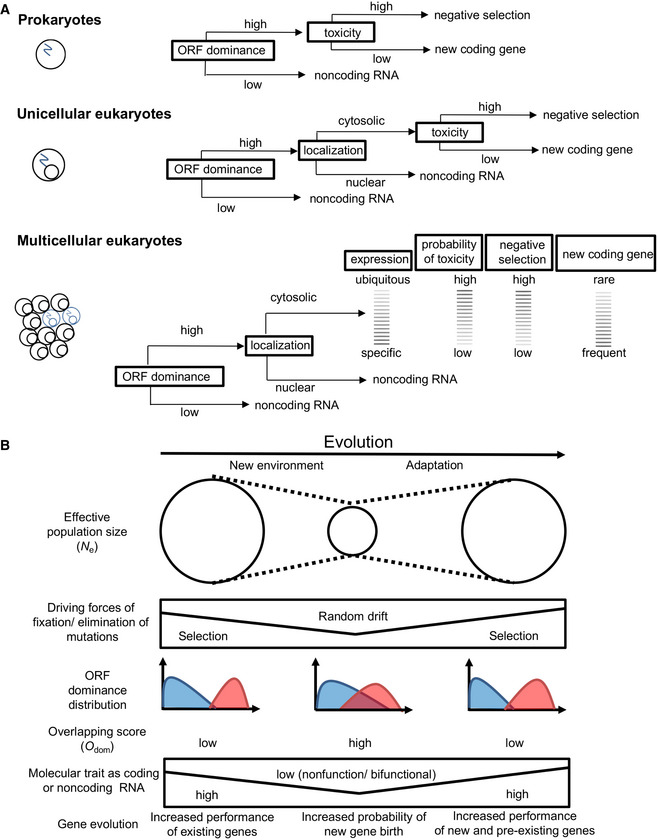
Hypothesis: gene birth is a countermeasure to the decline in effective population size Scheme explaining how nuclear evolution and multicellularity may contribute to the generation of noncoding RNAs with high ORF dominance in eukaryotes.Scheme illustrating new gene birth in response to the decline in effective population size caused by environmental changes.

In bacteria and archaea, newly transcribed RNAs are immediately bound by ribosomes (Miller *et al*, 1970; French *et al*, 2007) and cannot escape translation. Thus, as expected, lncRNAs in bacteria and archaea showed low ORF dominance (Fig 9A, top panel). Alternatively, in eukaryotes, the nucleus prevents the immediate binding of lncRNAs by ribosomes, and cytoplasmic translocation from the nucleus is required for translation. Therefore, eukaryotic lncRNAs may exist in the nucleus even with high ORF dominance, and the subsequent evolution of cytosolic translocation for these noncoding RNAs may contribute to the formation of new coding genes (Fig 9A, middle panel). Thus, the pervasive transcription of the genome seems to help eukaryotes to produce new noncoding/coding RNAs, while being disadvantageous for bacteria and archaea by increasing the risk of transcription of high ORF dominance transcripts, leading to immediate translation of wasteful and/or toxic proteins (Fig 9A, top and middle panels; Monsellier & Chiti, 2007). In addition, multicellular organisms have a variety of intracellular environments because of the large number of cell types, which may increase the probability of an intracellular environment in which newly originated proteins are not toxic (Fig 9A, bottom panel). As the probability that a new protein will not be toxic in multiple intracellular environments is lower than the probability that it will not be toxic in a particular intracellular environment, noncoding RNAs that are ubiquitously expressed need to have lower ORF dominance than those with specific expression (Fig 9A, bottom panel).

Kaessmann proposed an “out‐of‐the‐testis hypothesis”, arguing that testes facilitate the birth and evolution of new genes in animals (Kaessmann, 2010). His group has shown that germ cells (spermatocytes and spermatids) in the testes have an active chromatin state and have widespread transcriptional activity, resulting in the transcription of RNAs without immediate functional relevance (Soumillon *et al*, 2013). They discussed that this pervasive transcription increases the probability of generating new coding genes. Consistent with this hypothesis, our results showed that the ORF dominance distribution of noncoding RNAs shifted to higher values only in mature testes with spermatocytes and spermatids, but not in immature testes or other tissues. Wang *et al* (2020) recently identified cluster I genes that escape the global translation repression in spermatocytes and spermatids instead showing high translational efficiency (Wang *et al*, 2020), and we found that cluster I genes showed high ORF dominance. Therefore, new coding genes seem to be generated from transcripts with high ORF dominance that are specifically expressed in spermatocytes and spermatids. In support of this hypothesis, a recent study identified new functional *de novo‐*evolved proteins that regulate chromatin condensation in spermatids (Rivard *et al*, 2021).

Functional annotation of high ORF dominance noncoding transcripts was related to transcriptional regulation, and the target genes of transcription factors, including MYCN, TGIF, and ZIC2, were enriched. Notably, both NCYM and MYCN are expressed in germ cells of the testes (Suenaga *et al*, 2014; Kanatsu‐Shinohara *et al*, 2016), and MYCN has been shown to regulate the self‐renewal of spermatogonial stem cells (Kanatsu‐Shinohara *et al*, 2016). Furthermore, a recent study showed that binding sites for transcription factors, including MYCN, are mutational hot spots in human spermatogonia (Kaiser *et al*, 2021). Both TGIF and ZIC2 are mutated in holoprosencephaly, a disorder caused by a failure in embryonic forebrain development (Brown *et al*, 1998; Gripp *et al*, 2000), whereas MYCN mutations cause Feingold and megalocephaly syndromes, which are associated with reduced and increased brain size, respectively (van Bokhoven *et al*, 2005; Kato *et al*, 2019). Thus, the present study also provides a list of candidate human *de novo* genes possibly involved in brain development and brain‐related diseases.

According to the drift‐barrier hypothesis (Lynch, 2010; Lynch *et al*, 2016), the performance of any molecular trait is expected to become more refined in larger population sizes, because the effects of selection relative to random drift are stronger than in small populations. Consistent with this hypothesis, we found that the molecular traits of coding or noncoding RNAs were prominent in bacteria/archaea and weak in multicellular eukaryotes, allowing the existence of bifunctional or nonfunctional RNAs. The excessive overlap of ORF dominance distributions (*O*_dom_ > 0.7) diminished the correlation between ORF dominance and protein‐coding potential. This indicates that both coding and noncoding transcripts lost their molecular traits as coding and noncoding RNAs in terms of ORF dominance, which became lethal or highly deleterious for the species, probably because of the accumulation of nonfunctional RNAs.

Species with decreasing population sizes showed significantly higher *O*_dom_ than species with a stable population size, even those classified as LC in the IUCN Red List. Combined with the results discussed above, we propose a novel model for gene origin in which new gene birth occurs in response to decreased effective population sizes (Fig 9B). At stable population sizes, natural selection maintains the molecular traits of existing genes, and thus, the coding and noncoding functions of RNA stably coexist with high and low ORF dominance and low overlap of the ORF dominance distributions of coding and noncoding transcripts. When new environments reduce the effective population size of species, the driving force of fixation/elimination of mutations changes from natural selection to random drift. This increases the probability of fixation of neutral or slightly deleterious mutations (Kimura, 1968, 1983; Ohta, 1973), resulting in an increase in the overlap of ORF dominance distributions between coding and noncoding transcripts. This overlap allows the existence of nonfunctional or bifunctional RNAs as candidates for new coding or noncoding transcripts. Driven by random drift, the emergence of functions for these new transcripts is largely stochastic rather than shaped by selection, as observed for the novel ORFs in human lineages (Dowling *et al*, 2020).

When the effective population size approaches 1,000 because of rapid decline, the accumulation of deleterious mutations decreases the long‐term evolutionary potential of populations (Frankham *et al*, 2014), leading to extinction. On the contrary, when the speed is slow enough for the stochastically evolved new coding/noncoding transcripts to contribute to an increase in the effective population size, the species adapt to new environments. The increase in the effective population size leads to an increase in the effect of natural selection on the new functions of coding/noncoding genes and on those of preexisting genes.

In conclusion, ORF dominance is an important indicator for integrating the concept of gene birth into classical evolutionary theory, thereby contributing to the elucidation of the molecular basis for the evolution of complex species, including humans. In the future, it will be necessary to calculate ORF dominance based on the transcriptomes of additional species to test our hypothesis that positions new gene birth as a countermeasure to the decline in effective population size.

## Materials and Methods

### Primary and secondary ORFs

In this study, ORFs were defined as sequence segments beginning at AUG and ending with any of the UAA, UAG, or UGA stop codons in the 5ʹ to 3ʹ direction within an RNA sequence in all three possible reading frames (Fig 1A). The ORFs in the human *de novo* gene *NCYM* (Suenaga *et al*, 2014) were identified using its cDNA sequence (Appendix Fig S1A) and are shown in bold characters (Appendix Fig S1B). Sequences that begin at AUG and end at the 3ʹ‐terminus of RNA without UAA, UAG, or UGA were not considered ORFs. Hence, an RNA sequence lacking the AUG or the three base sequences that constitute stop codons (UAA, UAG, or UGA) did not contain ORFs. We did not use the reverse complement sequences of RNA sequences registered in databases to define ORFs because ribosomes translate mRNAs in the 5′ to 3′ direction.

### ORF length

#### Definition

The ORF length is defined as the length of the amino acid sequence, excluding the stop codon, and it is represented by *l* (Fig 1A). In an RNA sequence, the longest ORF is designated as the primary ORF (pORF), whereas the others are termed secondary ORFs (secORFs). The lengths of pORF and secORF are described as *l*_pORF_ and *l*_secORF_, respectively (Fig 1A). We excluded lncRNAs with pORFs shorter than 20 amino acids from our analyses because the existence or physiological significance of such short peptides is not clear in most of the species analyzed in the present study.

#### Example

The shortest possible ORF was “AUGUAA”, “AUGUAG”, or “AUGUGA”, with a single methionine. For example, the NCYM transcript has a pORF with a length of 109 in frame 1, three secORFs with lengths of 69, 8, and 6, respectively, in frame 2, and no ORFs in frame 3 (Appendix Fig S1C and D).

#### Characteristics

Therefore, the lengths of pORF and secORF present the following relationship:

(1) $$1\leq l_{\text{secORF}}\leq l_{\text{pORF}}$$

### ORF dominance

#### Definition

We defined ORF dominance (Fig 1A) according to Equations 2 and 3

(2) $$\sum_{i=1}^{n}l_{\text{secORF}i}=l_{\text{secORF}1}+l_{\text{secORF}2}+\cdots l_{\text{secORF}k}+\cdots +l_{\text{secORF}n}$$

(3) $$\text{ORF}\;\text{dominance}=\frac{l_{\text{pORF}}}{l_{\text{pORF}}+\sum_{i=1}^{n}l_{\text{secORF}i}},$$

where $l_{\text{pORF}}+\sum_{i=1}^{n}l_{\text{secORF}i}$ represents the sum of all ORF lengths.

This definition derived from the hypothesis that the potential for translation of a pORF is reduced by the translation of secORFs.

#### Example

For an RNA sequence with only one ORF, ORF dominance was 1 (Fig 1B). An RNA sequence with many secORFs tended to have a score close to 0 (Fig 1B). If the sum of all secORF lengths was equal to the pORF length, the ORF dominance was 0.5 (Fig 1B). The ORF dominance of the *NCYM* transcript was 0.568 (Appendix Fig S1C). In some transcripts, multiple ORFs have the longest length, causing the definition of pORF and secORF to become unclear. However, this was resolved by defining ORF dominance using only the sum of all ORF lengths $l_{\text{pORF}}+\sum_{i=1}^{n}l_{\text{secORF}i}$ in the denominator, and the length of the pORF to calculate ORF dominance. Therefore, ORF dominance is uniquely calculated, even for transcripts for which the pORF cannot be clearly defined. If an RNA sequence does not contain an ORF, both the numerator and denominator are set to 0. In such transcripts, there is no protein‐coding potential, and ORF dominance is not defined. Transcripts without ORFs were excluded from the analyses.

#### Characteristics

Therefore, the range of the ORF dominance is as follows:

(4) 0<ORFdominance≤1

### Relative frequencies *f*(*x*) and *g*(*x*)

#### Definition

We defined *f*(*x*) and *g*(*x*) according to Equations 5 and 6, respectively (Fig 1C):

(5) $$f\left(x\right)=\frac{NM(x)}{\mathrm{TNM}}$$

(6) $$g\left(x\right)=\frac{NR(x)}{\mathrm{TNR}},$$

where *TNM* and *TNR* represent the total numbers of coding and noncoding transcripts, respectively, excluding transcripts lacking ORFs. *NM*(*x*) and *NR*(*x*) are the numbers of coding and noncoding transcripts with an ORF dominance of *x*, respectively.

To define coding/noncoding transcripts with an ORF dominance of *x*, we divided the histograms into ten classes and used the median values of the classes to represent ORF dominance (Fig 1C). Therefore, in Equations 5 and 6, ORF dominance *x* was restricted as follows:

(7) x=0.05,0.15,0.25,0.35,0.45,0.55,0.65,0.75,0.85,or0.95

#### Characteristics

Thus, *f*(*x*) and *g*(*x*) follow Equations ((8), (9), (10), (11)):

(8) 0≤f(x)≤1

(9) 0≤g(x)≤1

(10) $$\sum_{x}f(x)=1$$

(11) $$\sum_{x}g(x)=1$$

### Overlapping scores *O*_dom_ and *O*_cov_

#### Definition

*O* (*x*) was calculated according to Equation (12):

(12) $$O\left(x\right)=\sum_{x}o(x),$$

where *o* (*x*) is the smaller value of the relative frequency of *f*(*x*) or *g*(*x*). *O*_dom_ is *O* (*x*) with ORF dominance = *x*, and *O*_cov_ is *O* (*x*) with ORF coverage = *x*.

### Protein‐coding potential *F*(*x*)

#### Definition

*F* (*x*) was calculated according to Equation (13):

(13) $$F\left(x\right)=\frac{f(x)}{f(x)+g(x)}$$

#### Example

For example, *F*(0.15) in human transcripts is shown in Fig 1D. *F*(0.15) was calculated using Equation (13), as follows:

f(0.15)=0.060

g(0.15)=0.268

$$F\left(0.15\right)=\frac{f(0.15)}{f(0.15)+g(0.15)}=\frac{0.060}{0.060+0.268}=0.18292=0.183$$

### Identification of noncoding transcripts with high protein‐coding potential

NR transcripts with high *F*(*x*) (0.6 ≤ *x* < 0.8) were identified from the total NR transcripts from the NCBI nucleotide database. NR transcripts shorter than 200 nucleotides or with pORFs encoding putative peptides with less than 20 amino acids were excluded. The amino acid sequences of pORFs in these transcripts were subjected to a BLASTP search to detect the presence of putative domain structures. In the BLASTP search, nonredundant protein sequences (nr) were applied as the search set, and quick accelerated protein–protein BLAST (BLASTP) was chosen as the algorithm. In the search results, putative conserved domains or the message “No putative conserved domains have been detected” were shown in the Graphical Summary tab. CDSEARCH/cdd was used to search for conserved domain structures using the default settings: low‐complexity filter, no; composition‐based adjustment, yes; E‐value threshold, 0.01; and maximum number of hits, 500. Based on these data, transcripts with or without putative conserved domain structures were indicated as + or −, respectively.

### Functional annotation of original genes

Original genes were defined as those noted in the official gene name of NR transcripts, including sense genes for antisense transcripts, homologous genes for pseudogenes, coding genes for noncoding transcript variants, and readthrough, divergent, or intronic transcripts. For lincRNAs, microRNA host genes, small nuclear RNAs, and other lncRNAs, the official gene symbol was used for annotation. This information was manually checked using the information available in the nucleotide database. DAVID (https://www.david.ncifcrf.gov) was used to identify the enriched molecular functions and pathways related to the original genes. *Q*‐values (*P*‐values adjusted for false discovery rate) were calculated using the Benjamini–Hochberg method in DAVID.

### *K*a‐to‐*K*s nucleotide substitution ratios

To identify orthologous regions between human transcripts and chimpanzee/mouse genomes, the BLAST‐like alignment tool (BLAT) v. 36 (Kent, 2002) was used for querying human transcript sequences with the estimated ORF dominance against chimpanzee (PtRV2) and mouse (GRCm38.p6) genomic sequences in the NCBI database. We defined the BLAT best‐hit genomic regions of chimpanzee/mouse as orthologs for each human transcript. The human–chimpanzee (or human–mouse) sequences were aligned for each exon region, and the sequences were combined for each transcript. Only orthologous sequence pairs of more than 60 bp in length (encoding > 20 amino acid residues) were extracted. *K*a and *K*s nucleotide substitution rates were estimated as described by Yang and Nielsen (2000) and implemented in PAML version 4.8a (Yang, 1997). Transcripts with high *K*a (> 1) or high *K*s (> 1) were excluded from our dataset as outliers. We calculated *K*a and *K*s for 47,228 NM human–chimpanzee, 14,116 NM human–mouse, 8,810 NR human–chimpanzee, and 1,561 NR human–mouse pairs.

### Relative frequencies of negatively selected genes

We defined the frequency of negatively selected genes, *h*(*x*), in both coding and noncoding transcripts (Fig 1G), as shown in Equation (14):

(14) $$h\left(x\right)=\frac{Nns(x)}{TNor(x)}$$

where *TNor* (*x*) represents the total number of coding or noncoding transcripts with orthologous sequences at ORF dominance = *x*. *Nns*(*x*) is the number of coding or noncoding transcripts with *K*a/*K*s < 0.5 at ORF dominance = *x*. The ORF dominance *x* was restricted as shown in Equation 7.

### Phylogenic trees

TimeTree (Hedges *et al*, 2006) was used to draw trees using official species names.

### Selection of viruses and identification of vORFs

The complete genomes of (+) ssRNA viruses infecting human or bacteria (Datasets EV14 and 15) were collected from the NCBI Virus database (Hatcher *et al*, 2017). vORFs were identified, and the sums of the vORF lengths $\sum_{i=1}^{n}l_{\text{vORF}i}$ were manually calculated. We eliminated those viruses that translated viral proteins after splicing or using exceptional translation mechanisms such as ribosome frameshifting, alternative initiation sites, ribosome slippage, and RNA editing.

### vORF score

#### Definition

The vORF score was calculated according to Equations ((15), (16), (17)):

(15) $$\sum_{i=1}^{n}l_{\text{vORF}i}=l_{\text{vORF}1}+l_{\text{vORF}2}+\cdots +l_{\text{vORF}k}+\cdots +l_{\text{vORF}n}$$

(16) $$\sum_{i=1}^{n}l_{\text{secORF}i}=l_{\text{secORF}1}+l_{\text{secORF}2}+\cdots +l_{\sec\text{ORF}k}+\cdots +l_{\sec\text{ORF}n}$$

(17) $$\text{vORF}\;\text{score}=\frac{\sum_{i=1}^{n}l_{\text{vORF}i}}{\sum_{i=1}^{n}l_{\text{vORF}i}+\sum_{i=1}^{n}l_{\text{secORF}i}},$$

where $l_{\text{vORF}i}$ represents the length of the bona fide ORFs, and $\sum_{i=1}^{n}l_{\text{secORF}i}$ is the sum of secORF lengths. $\sum_{i=1}^{n}l_{\text{vORF}i}+\sum_{i=1}^{n}l_{\text{secORF}i}$ represents the sum of the lengths of all ORFs.

### ORF dominance calculations using transcriptome data

Transcriptome data of five species were obtained from a previous study (Sarropoulos *et al*, 2019). Ensembl transcript IDs of cluster I and cluster III genes were obtained from the authors of a previous study (Wang *et al*, 2020). All transcripts expressed at detectable levels (nonzero) in each tissue were used to calculate ORF dominance for lncRNAs and to plot ORF dominance distributions. To determine the correlation between tissue specificity and ORF dominance, we divided the transcripts into the indicated groups according to the number of tissues in which the transcript was detected and described the ORF dominance distribution in each group. Human transcriptome data for coding transcripts were obtained from the Human Protein Atlas (http://www.proteinatlas.org), including RNA isoform data from 131 cell lines and 281 tissues. The ORF dominance for each transcript was calculated from Ensembl data.

### Statistical analyses

Statistical analyses were performed using Excel and R software (R Project for Statistical Computing, Vienna, Austria).

## Author contributions

**Yusuke Suenaga:** Conceptualization; Data curation; Formal analysis; Supervision; Funding acquisition; Validation; Investigation; Visualization; Methodology; Writing—original draft; Project administration; Writing—review & editing. **Mamoru Kato:** Conceptualization; Data curation; Supervision; Validation; Investigation; Methodology; Writing—original draft; Project administration; Writing—review & editing. **Momoko Nagai:** Data curation; Software; Formal analysis; Validation; Methodology; Writing—original draft; Writing—review & editing. **Kazuma Nakatani:** Data curation; Formal analysis; Validation; Investigation. **Hiroyuki Kogashi:** Data curation; Formal analysis; Validation; Investigation. **Miho Kobatake:** Data curation; Formal analysis; Validation; Investigation. **Takashi Makino:** Data curation; Formal analysis; Supervision; Methodology; Writing—original draft.

In addition to the CRediT author contributions listed above, the contributions in detail are:

YS conceived and developed the research plan. YS, MKa, MN, KN, HK, MKo, and TM analyzed the data. YS, MKa, and TM wrote the manuscript.

## Disclosure and competing interests statement

The authors declare that they have no conflict of interest.

## Supporting information



Appendix

Dataset EV1

Dataset EV2

Dataset EV3

Dataset EV4

Dataset EV5

Dataset EV6

Dataset EV7

Dataset EV8

Dataset EV9

Dataset EV10

Dataset EV11

Dataset EV12

Dataset EV13

Dataset EV14

Dataset EV15

## Data Availability

The source data for statistical analyses and figures (10 example datasets) are available on https://doi.org/10.6084/m9.figshare.7269500. The code associated with generating and analyzing these tables is available on https://doi.org/10.6084/m9.figshare.7269518.
