# Supplementary material for: Open reading frame dominance indicates protein‐coding potential of RNAs
Source: EMBO Rep. 2022 Apr 19;23(6):e54321. doi: 10.15252/embr.202154321 (PMC9171421; doi:10.15252/embr.202154321)
Supplement: Supplementary file 1 — Appendix [file EMBR-23-e54321-s002.pdf]

Appendix Figures

Open reading frame dominance indicates protein-coding potential of RNAs

Yusuke Suenaga[1,‡*], Mamoru Kato[2,‡], Momoko Nagai[2], Kazuma Nakatani[1,3,4], Hiroyuki Kogashi[1,3], Miho Kobatake[1], Takashi Makino[5]

[1]Department of Molecular Carcinogenesis, Chiba Cancer Centre Research Institute, 666-2 Nitona, Chuo-ku, Chiba 260-8717, Japan

[2]Division of Bioinformatics, National Cancer Centre Research Institute, 5-1-1 Tsukiji, Chuo-ku, Tokyo 104-0045, Japan

[3]Department of Molecular Biology and Oncology, Chiba University School of Medicine, 666-2 Nitona, Chuo-ku, Chiba 260-8717, Japan

[4]Innovative Medicine CHIBA Doctoral WISE Program, Chiba University School of Medicine, 666-2 Nitona, Chuo-ku, Chiba 260-8717, Japan

[5]Laboratory of Evolutionary Genomics, Graduate School of Life Sciences, Tohoku University, Aobayama Campus 6-3, Aramaki Aza-Aoba, Aoba-ku, Sendai 980-8578, Japan

[‡]These authors contributed equally to this work

***Correspondence to:**

Yusuke Suenaga

E-mail: ysuenaga@chiba-cc.jp

Table of contents

Appendix Figure S1 - ORFs of *NCYM* and an example of ORF dominance calculation……………..3

Appendix Figure S2 - Relationships between the relative frequencies of coding and noncoding transcripts for human and mouse ORF dominances………………………………………………. 4

Appendix Figure S3 - ORF dominances ≤ 0.65 correlate with protein-coding potential, $F(x)$, in human and mouse transcripts………………………………………………………………………5

Appendix Figure S4 - Relationships between ORF dominance and the relative frequencies of coding and noncoding transcripts in archaea…………………………………………………………6

Appendix Figure S5 - Relationships between ORF dominance and the relative frequencies of coding and noncoding transcripts in plants…………………………………………………...……………7

Appendix Figure S6 - Relationships between ORF dominance and the relative frequencies of coding and noncoding transcripts in the species shown in Figure 6………………………………………8

Appendix Figure S7 - Relationship between the overlaps of ORF coverage distributions and effective population sizes……………………………………………………………………...……………...9

Appendix Figure S8 - Relationship between ORF dominance and protein-coding potential, $F(x)$, for 32 eukaryotes……………………………………………………………………………………...10

Appendix Figure S9 - ORF dominance distributions of lncRNAs in tissues from four mammals….11

Appendix Figure S10 - Relationships between tissue specificity and ORF dominance distributions for noncoding transcripts from four mammals…………………………………………………...12

Appendix Figure S11 - Relationships between tissue-specificity and ORF dominance distributions for coding transcripts………………………………………………………………………………13

A

>NR_161162.1 Homo sapiens MYCN opposite strand (MYCNOS),
transcript variant 2, long non-coding RNA
CATTTCATTCACACAAGGCACTGCCTGGGGGAGGGGGCTGTTCCTGGCTGCAGAATTCTAGCTCTCACGA
GCACGCAGACAACCGCACTCGCAGCGGTGTGGGGCCGGCTGCTCAGGGGAAGCCCCAGGCTCTCCGACCC
AGCTACCGGGAATGGGGCACCCTTTGGAGAAGAACCCCAGCCTGGGGTGGGGACGCACCGGCTCTCCGAC
AGCTCAAACACAGACAGATCTTCTAGAGCCGAGGGAATTTCTTTTCGCAGAAGCCATTACTCCCCCCGAG
AGAAGGCTGCAAAGCTGGGAAGCCCAGGGTGTGCTCCTCCCGCCCTTTTGGACCCCCGGGCTTGCACCGG
CTGCACTCTGAGAACCAGCTGCGCGCGGAGCGGTGCAATGCAGCACCCACCCTGCGAGCCTGGCAATTGC
TTGTCATTAAAAGAAAAAAAAATTACGGAGGGCTCCGGGGGTGTGTGTTGGGGAGGGGAGACCGATGCTT
CTAACCCAGCCCCCGCTTTGACTGCGTGTTGTGCAGCTGAGCGCGAGGCCAACGTTGAGCAAGGCCTTGC
AGGGAGGTTGCTCCTGTGTAATTACGAAAGAAGGCTAGTCCGAAGGTGCAAAATAGCAGGGAGAGGACGC
GCCCCCTTAGGAACAAGACCTCTGGATGTTTCCAGTTTCAAATTGAAAGAAGAGGGGCGCCCCCCTTGTT
TGAAAATAAATAAATAAATAAGTGCGAGCTAC

B

Sequence Name	ORF size	Ficket Score	Hexamer Score	Coding Probability	Coding label
NR_161162.1	330	0.504	-0.1207753	0.022153	no

C

5'3' Frame 1
HFIHTRHCLGEGAVPGCRILALTSTQTTALAAVWGRLLRGSPRLSDPATGNGAPFGEEPQPGV
GTHRLSDSSNTDRSSRAEGISFRRSHYSPREKAAKLGSPGCAPPALLDPRACTGCTLRTSCAR
SGAMQHPPCEPGNCLSLKEKKITEGSGGVCWGGETDASNPAPALTACCAAEREANVEQGLAGR
LLLCNYERRLVRRCKIAGRGRAPLGTRPLDVSSFKLKEEGRPPCLKINK *ISASY
5'3' Frame 2
ISFTQGTAWGRGLFLAAEF*LSRARRQPHSQRCGAGCSGEAPGSPTQLPGMGHPLEKNPSLGW
GRTGSPTAQTQTDLLEPREFLFAEAITPPERRLQSWEAQGVLLPPFWTPGLAPAAL*EPAARG
AVQCSTHPASLAIACH*KKKKLRRAPGVCVGEGRPMLLTQPPL*LRVVQLSARPTLSKALQGG
CSCVITKEG*SEGAK*QGEDAPP*EQDLWMFPVSN*KKRGAPLV*K*INK*VRA
5'3' Frame 3
FHSHKALPGGGGCSWLQNSSSHEHADNRTRSGVGPAAQGKPQALRPSYREWGTLWRRTPAWGG
DAPALRQLKHRQIF*SRGNFFSQKPLLPPREGCKAGKPRVCSSRPFGPPGLHRLHSENQLRAE
RCNAAPTLRAWQLLVIKRKKNYGGLRGCVLGRGDRCF*PSPRFDCVLCS*ARGQR*ARPCREV
APV*LRKKASPKVQNSRERTRPLRNKTSGCFQFQIERRGAPPLFENK*INKCEL

D

$$l_{\mathrm{pORF}} = 109$$

$$\sum_1^3 l_{\mathrm{secORF}k} = 69 + 8 + 6 = 83$$

$$\text{ORF dominace} = \frac{109}{109+83} = 0.568$$

$$F(x) = 1.301 \times 0.568 + 0.0072 = 0.746 \text{ (Ensemble)}$$

$$F(x) = 1.313 \times 0.568 + 0.0189 = 0.765 \text{ (RefSeq)}$$

Appendix Figure S1. ORFs of NCYM and an example of ORF dominance calculation.

(A) NCYM cDNA sequence.

(B) Coding prediction of NCYM by CPAT (http://lilab.research.bcm.edu).

(C) Translated amino-acid sequence of NCYM in the three frames in the 5′ to 3′ (sense) direction. Red characters, pORF; blue characters, secORFs. Stop codons are shown as asterisks.

(D) Calculation of ORF dominance and F(x) for the NCYM transcripts. The length of pORF is 109 and the sum of secORF lengths is 83; therefore, the ORF dominance is 0.568.

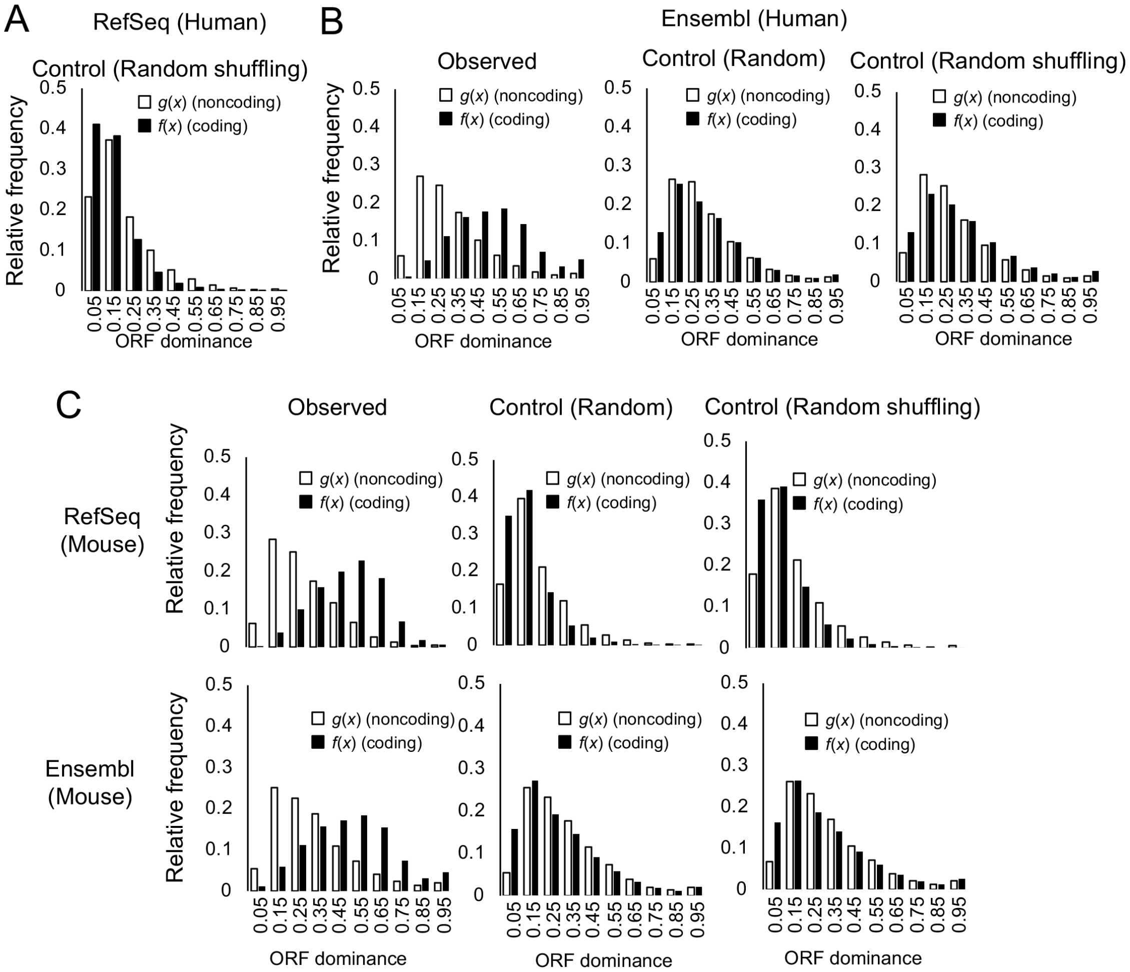

Appendix Figure S2. Relationships between the relative frequencies of coding and noncoding transcripts for human and mouse ORF dominances.

(A) Histogram of ORF dominance relative frequencies in coding, $f(x)$, and noncoding, $g(x)$, human transcripts with random shuffling controls using human data sets from RefSeq.

(B) Histogram of ORF dominance relative frequencies in coding, $f(x)$, and noncoding, $g(x)$, human transcripts with observed data (left), random controls (center), or random shuffling controls (right) using human data sets from Ensembl.

(C) Relative frequencies of coding, $f(x)$, and noncoding, $g(x)$, transcripts calculated using mouse data sets from RefSeq (upper panels) or Ensembl (lower panels).

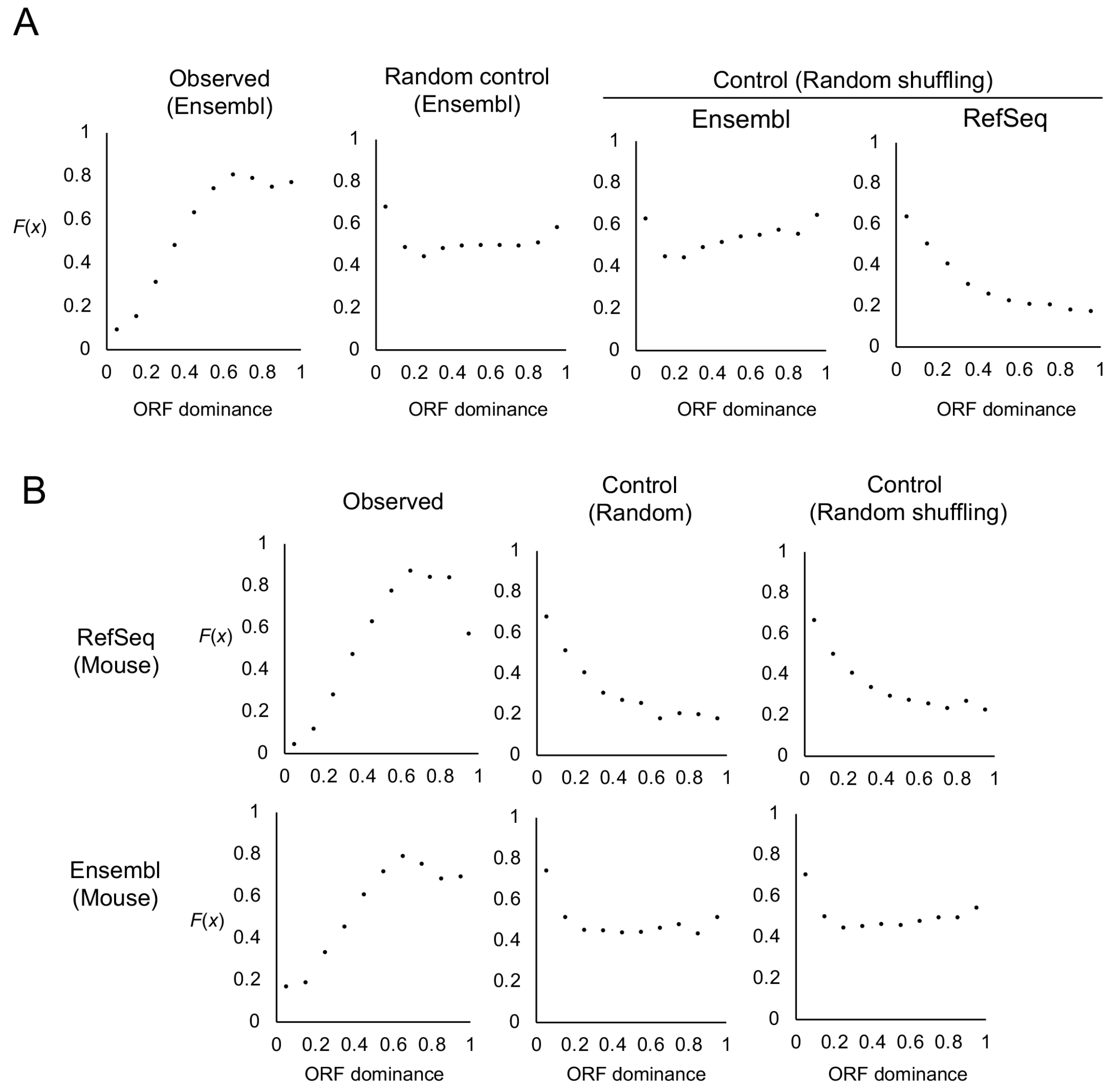

Appendix Figure S3. ORF dominances ≤ 0.65 correlate with protein-coding potential, *F*(*x*), in human and mouse transcripts.

(A) Relationship between ORF dominance and *F*(*x*) in a human data set from Ensemble and random controls (center). Randomly shuffled controls (right) were generated from human data sets from both Ensemble and RefSeq.

(B) Relationships between ORF dominance and *F*(*x*) in mouse transcripts using data sets from RefSeq (upper panels) or Ensembl (lower panels).

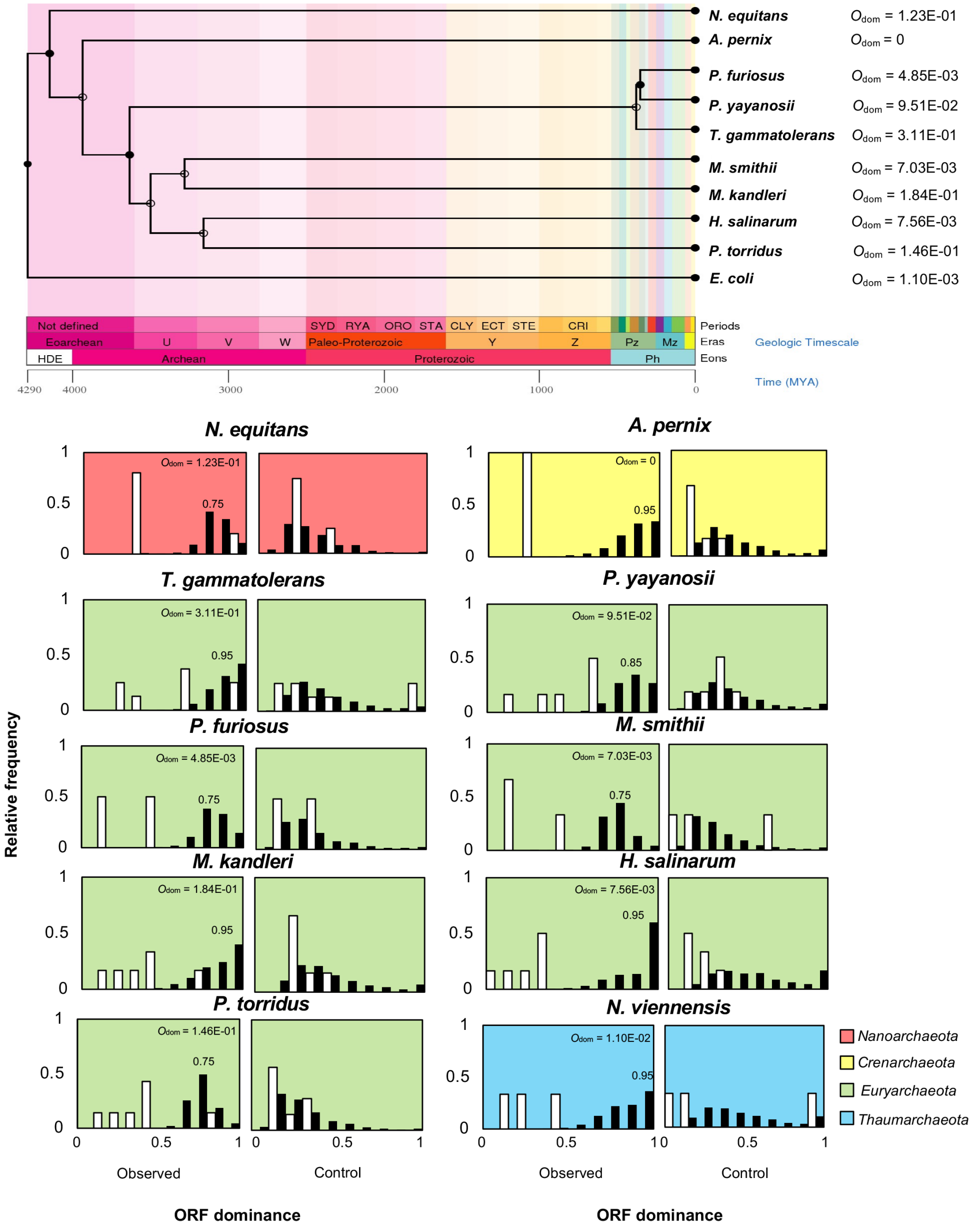

Appendix Figure S4. Relationships between ORF dominance and the relative frequencies of coding and noncoding transcripts in archaea. Phylogenetic tree for nine archaeal species and histogram of $f(x)$ (white) or $g(x)$ (black) in the observed data (left) and in nucleic-acid–scrambled controls (right). ORF dominances with highest $f(x)$ are indicated in the histograms. The lineage of one archaea species (*Nitrososphaera viennensis* EN76) is unknown and therefore it was excluded from the phylogenic tree. O_{dom} was calculated using the ORF dominance distribution of observed data.

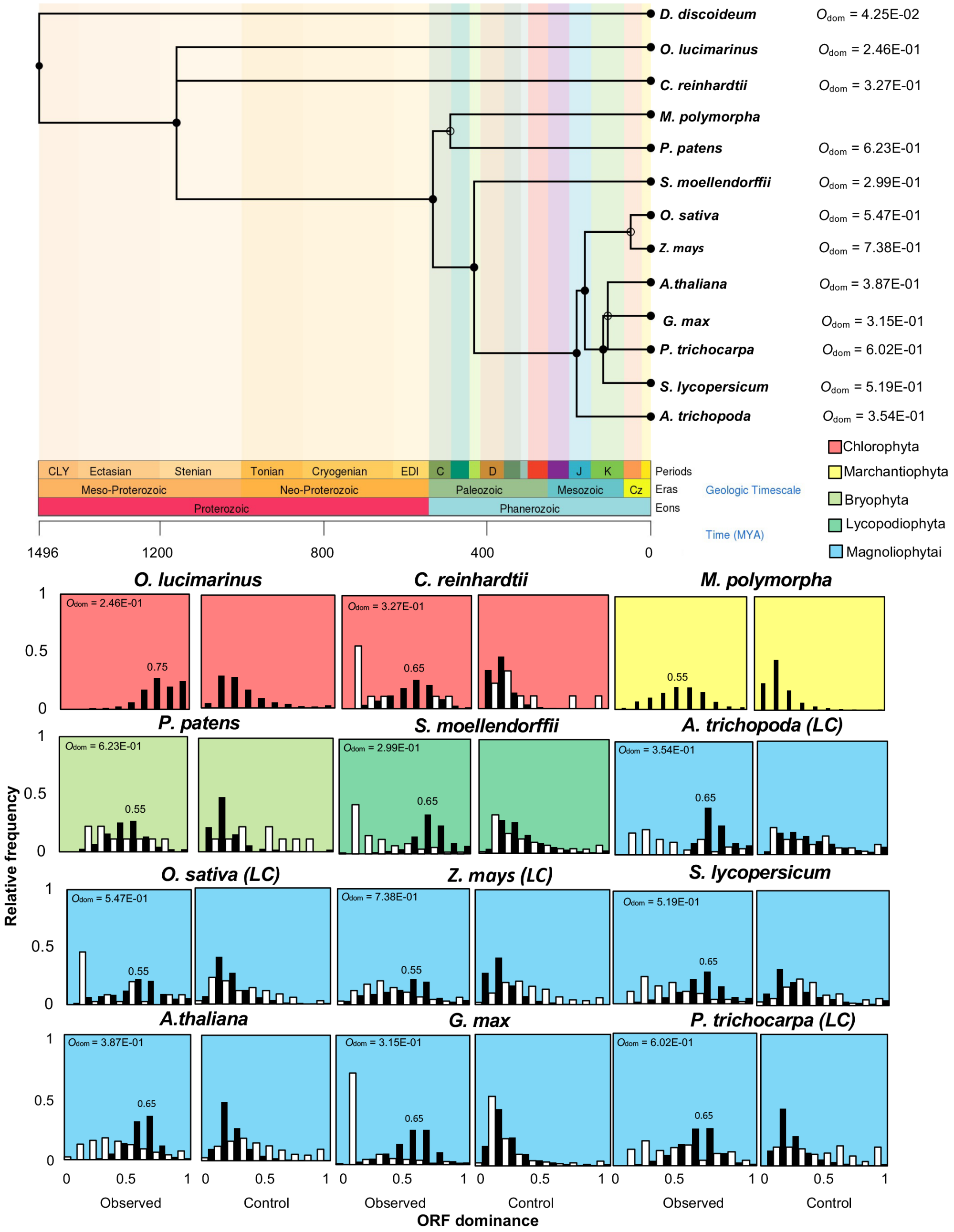

Appendix Figure S5. Relationships between ORF dominance and the relative frequencies of coding and noncoding transcripts in plants. Phylogenetic tree for 12 plants and histogram of $f(x)$ (white) or $g(x)$ (black) in the observed data (left) and in sequence–scrambled controls (right). ORF dominances with highest $f(x)$ are indicated. O_{dom} was calculated using the ORF dominance distribution of observed data.

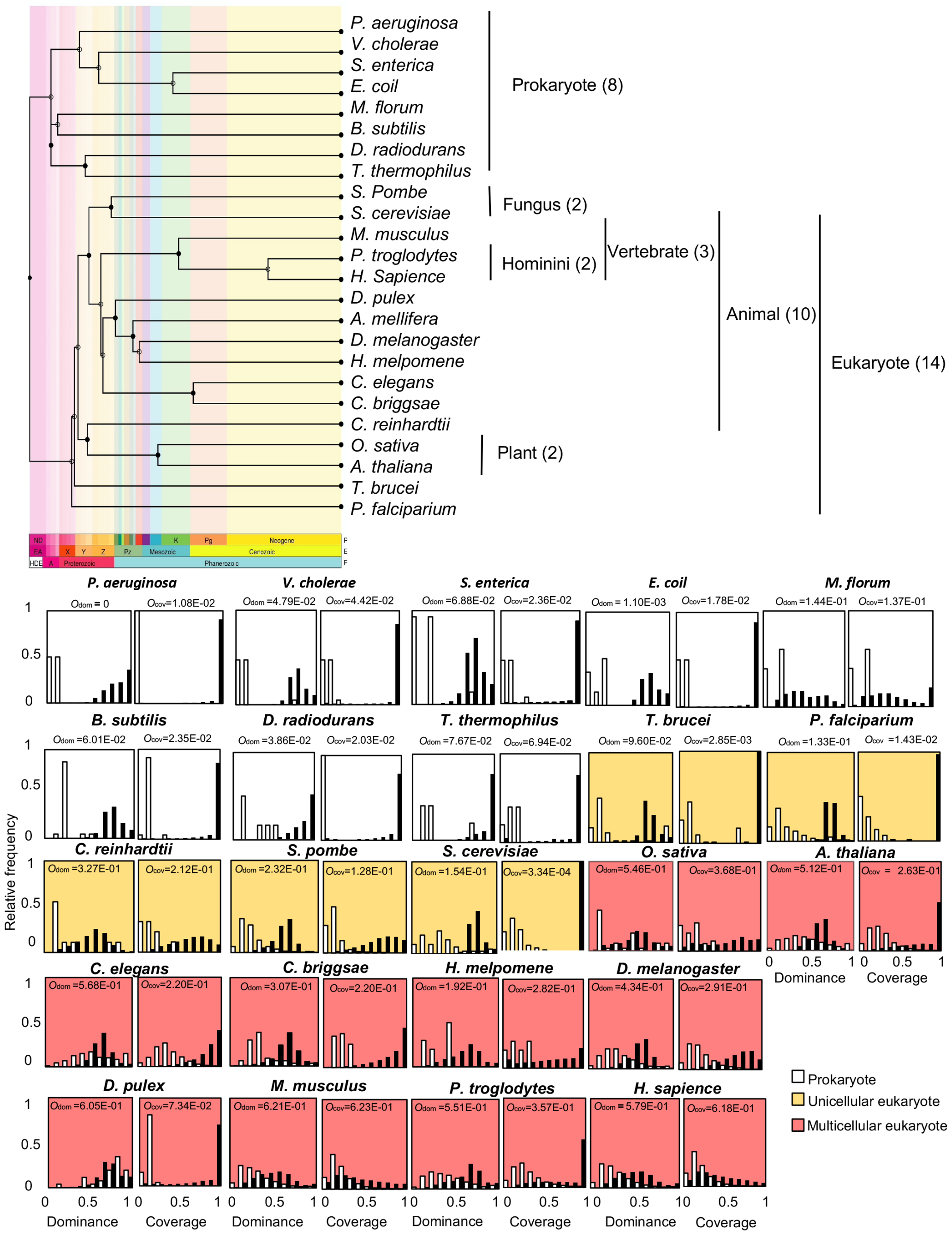

Appendix Figure S6. Relationships between ORF dominance and the relative frequencies of coding and noncoding transcripts in the species shown in Figure 6. Phylogenetic tree for 24 cellular organisms and histograms of $f(x)$ (white) or $g(x)$ (black) for ORF dominances (left) and ORF coverage (right). O_{dom} and O_{cov} were calculated using the distribution of observed data.

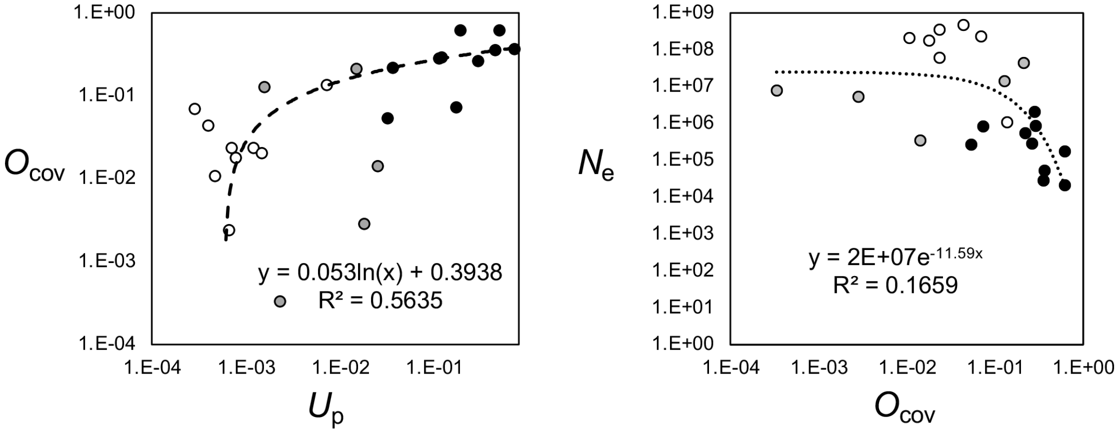

Appendix Figure S7. Relationship between the overlaps of ORF coverage distributions and effective population sizes. Dot plots of O_{cov} and U_p (left) or N_e (right). These relationships are approximately logarithmic or exponential, respectively. White, gray, and black dots indicate bacteria, unicellular eukaryotes, and multicellular eukaryotes, respectively.

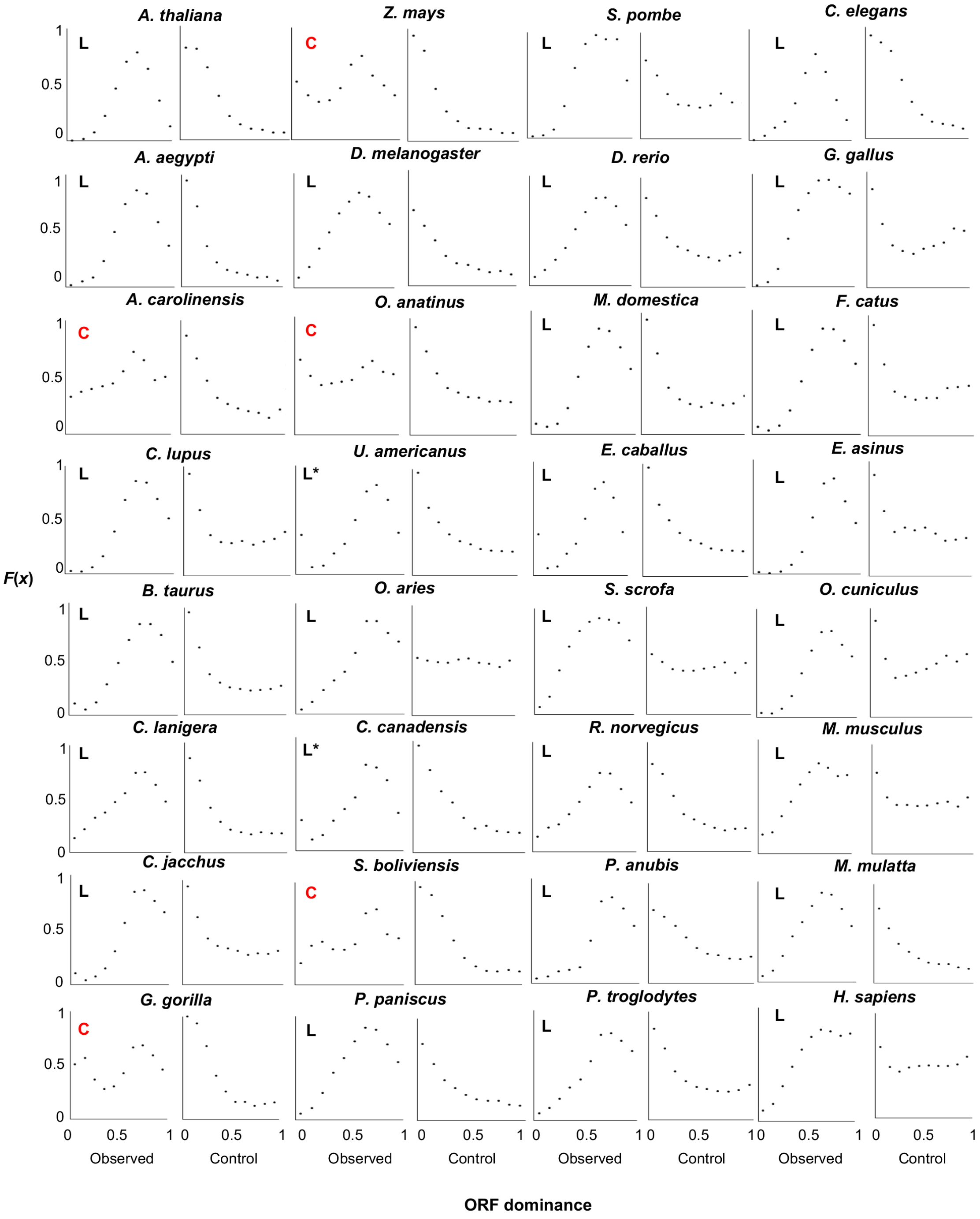

Appendix Figure S8. Relationship between ORF dominance and protein-coding potential, *F*(*x*), for 32 eukaryotes. Data sets from Ensembl (observed data, used in Figure 7) and random controls. Mouse and human data are identical to those shown in Supplementary Figure 3. Shapes of approximate functions are shown as L or C, indicating linear (in black) and constant (in red) functions, respectively. Numbers of lncRNAs with ORF dominance 0.05 were < 5 in *U. americanus*, *C. canadensis*, and *G. gorilla*. Therefore, we eliminated the *F*(0.05) from these species for the approximation by linear functions (asterisks).

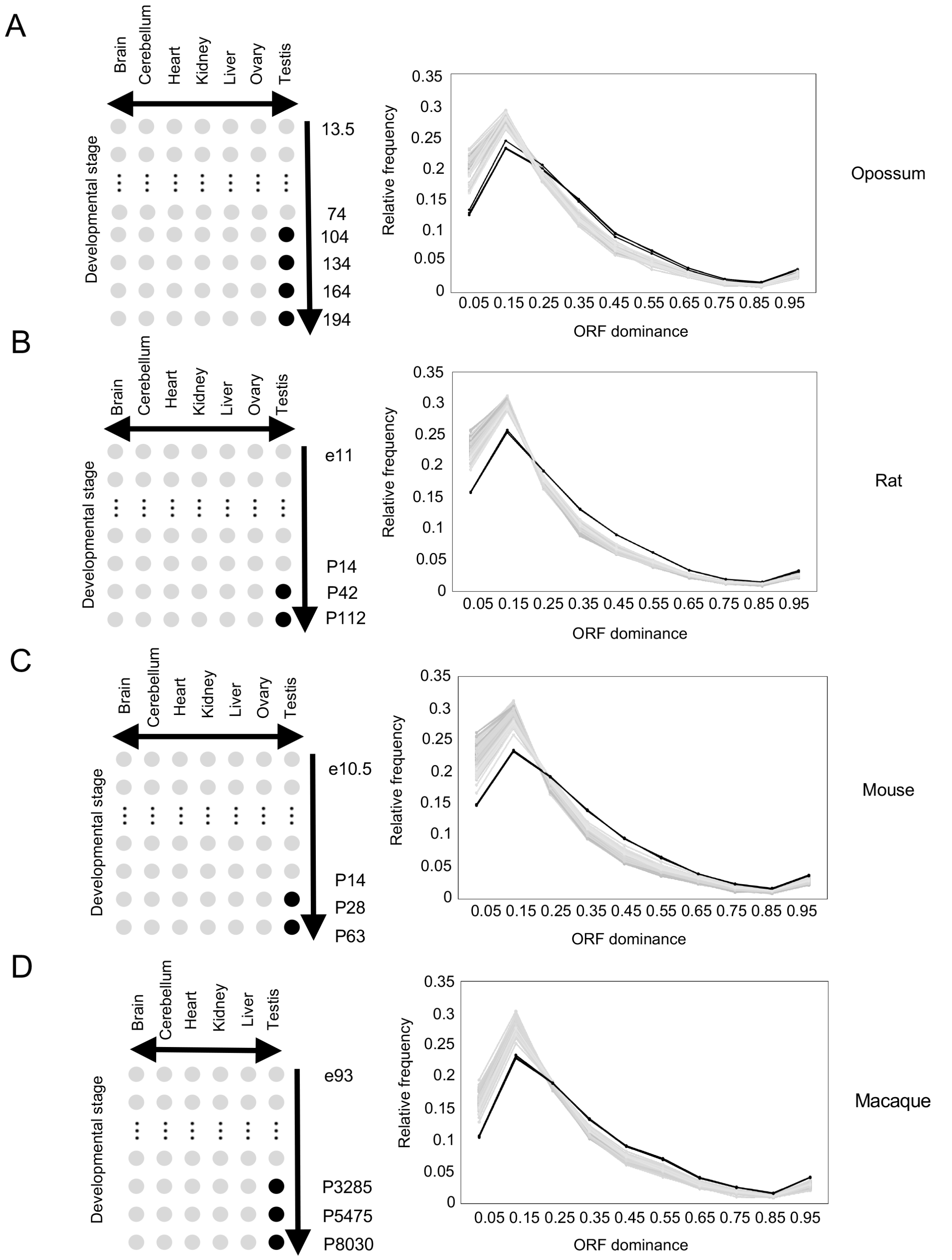

Appendix Figure S9. ORF dominance distributions of lncRNAs in tissues from four mammals. (A-D) Scheme illustrating of tissues used in calculation of ORF dominance (left). Mature testes and other tissues are indicated as black and gray circles, respectively. ORF dominance distributions (right) for mature testes and other tissues are indicated as black and gray lines, respectively.

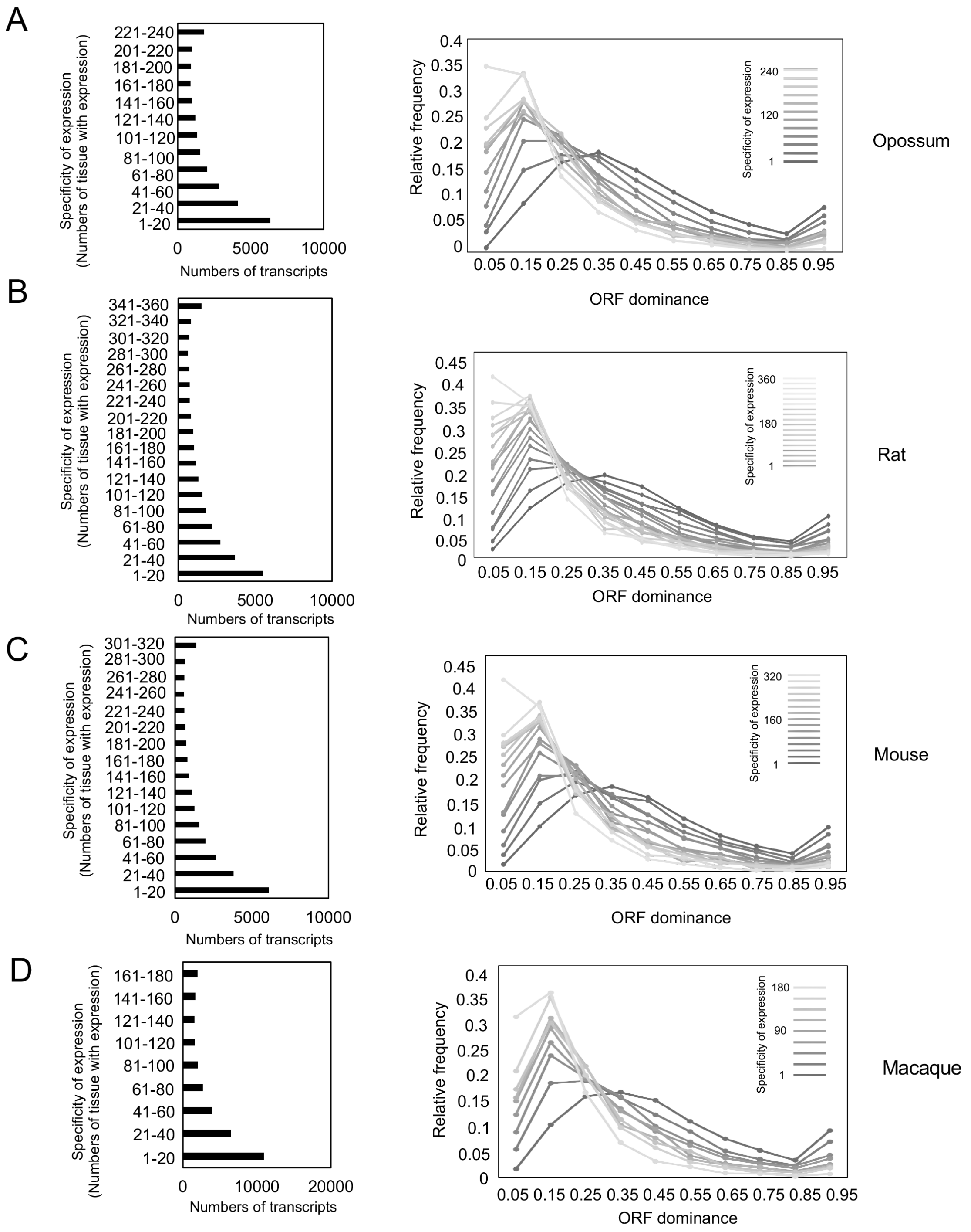

Appendix Figure S10. Relationships between tissue specificity and ORF dominance distributions for noncoding transcripts from four mammals.

(A-D) Relationship between specificity of expression and numbers of transcript (left). Transcripts with specific expression show high ORF dominance (right). Line intensity increases with increasing specificity of gene expression.

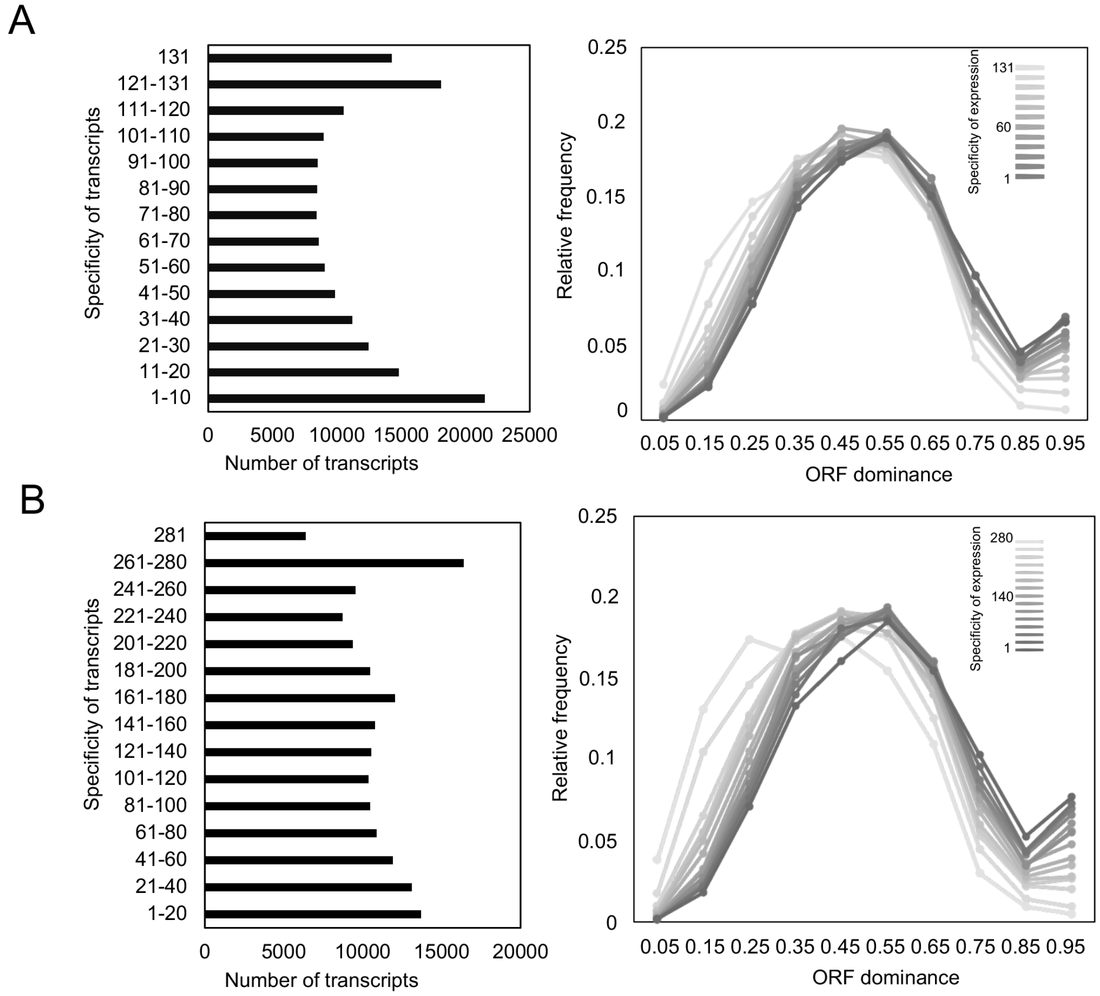

Appendix Figure S11. Relationships between tissue-specificity and ORF dominance distributions for coding transcripts

(A, B) Human cell lines (A) or tissues (B). Relationship between specificity of expression and numbers of transcript (left). Transcripts with specific expression show high ORF dominance (right). Line intensity increases with increasing specificity of gene expression.
