# Supplementary material for: Open reading frame dominance indicates protein‐coding potential of RNAs
Source: EMBO Rep. 2022 Apr 19;23(6):e54321. doi: 10.15252/embr.202154321 (PMC9171421; doi:10.15252/embr.202154321)
Supplement: Supplementary file 17 [file EMBR-23-e54321-s007.pdf]

EMBO reports

Open reading frame dominance indicates protein-coding potential of RNAs

Yusuke Suenaga, Mamoru Kato, Momoko Nagai, Kazuma Nakatani, Hiroyuki Kogashi, Miho Kobatake, and Takashi Makino
DOI: 10.15252/embr.202154321

Corresponding author(s): Yusuke Suenaga (ysuenaga@chiba-cc.jp)

Review Timeline:

Transfer from Review Commons:	12th Nov 21
Editorial Decision:	16th Dec 21
Revision Received:	15th Feb 22
Editorial Decision:	18th Mar 22
Revision Received:	24th Mar 22
Accepted:	25th Mar 22

Editor: Esther Schnapp

Transaction Report: This manuscript was transferred to EMBO Reports following peer review at Review Commons

(Note: With the exception of the correction of typographical or spelling errors that could be a source of ambiguity, letters and reports are not edited. Depending on transfer agreements, referee reports obtained elsewhere may or may not be included in this compilation. Referee reports are anonymous unless the Referee chooses to sign their reports.)

Review #1

1. How much time do you estimate the authors will need to complete the suggested revisions:

Estimated time to Complete Revisions (Required)

(Decision Recommendation)

Between 3 and 6 months

2. Evidence, reproducibility and clarity:

Evidence, reproducibility and clarity (Required)

Summary

The manuscript submitted by Suenaga and co-authors presents a method to evaluate the protein-coding potential of transcripts. This method is based on an index that they name the PTI (potentially translated island) score, which represents the ratio between the length of the largest predicted ORF and the sum of all the predicted ORF lengths, for each transcript. The author compare PTI score distributions between transcripts classified as protein-coding and as non-coding in public nucleotide databases, for a wide range of species, including bacteria, archaea, eukaryotes and viruses. They derive from this comparison a measure of the protein-coding potential of transcripts. To validate this approach, the authors evaluated the distributions of Ka/Ks values for transcripts annotated as coding or non-coding, in various classes of PTI-based protein-coding potential. The main finding of the manuscript stems from the comparison among species: the authors find that bacteria and archaea have narrow, non-overlapping PTI distributions for coding and non-coding transcripts, while eukaryotes have broader and more overlapping PTI distributions.

Major comments

- The authors provide no rationale for using the PTI score to measure the protein-coding potential of transcripts. The only attempt to justify this measure is given in the methods: "The definition of PTI score is motivated by our hypothetical concept that translation of pPTI is limited by alternate competing sPTIs." (lines 426-427, page 20). What the PTI score measures is the dominance of the largest predicted ORF over the predicted ORFs, in terms of length. It is not clear why there would be competition for translation of putative ORFs for genuine protein-coding transcripts. An alternative hypothesis, briefly touched upon in the discussion (lines 318-320) is that translation of non-functional ORFs could give rise to the production of toxic proteins, in addition to being costly in terms of energy. The authors should provide the reasoning behind the PTI score and should explain the biological mechanisms that may underlie differences between coding and non-coding transcripts.
- The presence of ORFs in transcripts has long been used as a predictor of their protein-coding potential. For

example, the ORF size and the ORF coverage are part of the set of predictors implemented in CPAT (Wang et al., 2013). The PTI score is necessarily related to these methods, yet no comparison is provided. If the PTI score is to be used as a measure to classify transcripts as coding or non-coding, its performance should be compared to other classifiers, including those that use the presence of ORFs as a predictor (e.g., CPAT) but not only (e.g., PhyloCSF, based on the pattern of sequence evolution).
- The authors compare the observed PTI score distributions with the PTI scores from random or shuffled sequences. They conclude that the PTI scores do not depend on transcript lengths but on transcript sequences (lines 122-123). However, this is not true for non-coding RNAs, for which the observed and randomized distributions are very similar. The relationship between transcript length and PTI scores should be analyzed into more detail. Are the annotated non-coding transcripts with high PTI scores particular in terms of length?
- The authors discuss in depth the correlation between PTI scores and PTI-based protein-coding potential measures (e.g., section "PTI scores correlate with protein-coding potential in humans and mice", starting line 125; section "Relationship between the PTI score and protein-coding potential", starting line 243). Given that the protein-coding potential is directly derived from the PTI score distributions for coding and non-coding transcripts, it is not surprising that the two should be correlated. The significance of observing a linear or a sigmoid relationship is not clearly explained.
- The authors use the entire set of annotated coding and non-coding transcripts to assess the distribution of PTI scores and to define the protein-coding potential. Traditionally, for methods that aim to classify transcripts as coding or non-coding, this is done using "bona fide" coding and non-coding transcripts, which are used as training sets. The efficiency of the method can then be evaluated using a test set of transcripts. This aspect is lacking here and should be implemented.
- The comparisons among species are likely biased by the quality of lncRNA annotations in non-model organisms - cf. high variations among primates, which are likely driven by the annotation quality and depth.
- The differences among bacteria, archaea and eukaryotes should be discussed into more depth. In bacteria, the genuine ORF is well defined by the presence of translation signals (e.g., Shine-Dalgarno sequence). Other factors are also at work in both prokaryotes and eukaryotes, including RNA secondary structures. The relationship between these factors and the PTI score should be discussed.
- From an evolutionary perspective, the effective population size (Ne) is also likely related to the "quality" of the ORFs. An analysis of Ne vs. the PTI score distributions would be an interesting addition to this manuscript.

3. Significance:

Significance (Required)

This manuscript is lacking in novelty and is not well positioned in the field. If the aim of this work is to provide a method to classify transcripts as coding or non-coding, the authors should provide detailed comparisons with existing methods (see above). If the aim is to understand what defines a genuine protein-coding transcript, then the biological mechanisms should be better described and the comparisons among species and among functional categories of genes should be further developed. The idea of using the "dominance" of the largest ORF compared to the other predicted ORFs is interesting, and provides a new element compared to existing methods that rely exclusively on ORF length and ORF coverage. I would recommend that the authors develop this idea further and discuss the advantages of using the ORF dominance compared to just the ORF length or coverage.

Referee Cross-commenting

I fully agree with Reviewer 2's remarks. In particular, adding ribosome profiling analyses is an excellent idea and could substantially improve the manuscript.

Review #2

1. How much time do you estimate the authors will need to complete the suggested revisions:

Estimated time to Complete Revisions (Required)

(Decision Recommendation)

Between 1 and 3 months

2. Evidence, reproducibility and clarity:

Evidence, reproducibility and clarity (Required)

In the manuscript "Potentially translated sequences determine protein-coding potential of RNAs in cellular organisms" Suenaga and colleagues analyze the available transcriptomes from 100 prokaryotes and eukaryotes, as well as >100 viruses to understand whether transcripts tend to be translated or not. They develop a potentially translated island score (PTI) that combines the number and length of open reading frames in a transcript. From there they develop a protein-coding potential score that combines PTI with database information on coding and non-coding transcripts in various organisms and that in some sense predicts whether a transcript would fall in the coding or non-coding category. The main takeaway appears to be that in prokaryotes PTIs and protein coding potential strongly differentiates coding and non-coding transcripts, while in eukaryotes these differences appear to be more fluid. The manuscript presents an interesting bioinformatic analysis of coding properties across the phylogenetic field and may represent an interesting resource. The audience for the manuscript at the moment may be more limited to scientists trained and working in the field of bioinformatics, but with some integration of transcriptomics and ribosome profiling data, as well as an effort to make the results accessible to scientists not trained in bioinformatics, this manuscript may be relevant and of interest to researchers working on the biology of long non-coding RNAs and translation in general.

Major comments:

- some validation of their predictions of coding potential would be good to add. There are plenty of ribosome profiling experiments out there for some of the studied organisms (human, mouse, E. coli) that could be used to show that indeed some of the non-coding RNAs are misclassified and have ribosome density across the predicted open reading frames.
- the manuscript is at times difficult to follow and the implication of the statements may not be immediately clear to the readers, particularly those without formal training in bioinformatic methods; even in the abstract. Some examples: "The relationship between the PTI score and protein-coding potential was sigmoidal in most eukaryotes; however,it was linear passing through the origin in three distinct eutherian lineages, including humans". Here it is not clear what this means (without reading the paper) - and even after reading the paper

the importance of noting the sigmoidal vs linear relationship of PTI vs. protein-coding potential is unclear. I would encourage the authors to double-check that they provide a clear interpretation of their results, with readers unschooled in proper statistics in mind.
- For the definition of PTI and protein-coding potential the authors refer to the Materials and Methods. I would encourage to explain in plain terms in the results section 1.) how they decided on this particular formalization and 2.) explain clearly what this means.
- The definition of protein coding potential for appears to be dependent on database classification of a transcript as either coding and non-coding. Particularly for organisms with complex transcriptomes, databases may not contain the proper information - what are the implications for their protein-coding potential score?
- The authors completely ignore plants - would it make sense to expand their analysis to this branch of the tree of life?

3. Significance:

Significance (Required)

The manuscript presents an elegant way to predict protein-coding and non-coding RNAs, which may be very relevant to the study of organisms with complex transcriptomes.

The audience for the manuscript at the moment may be more limited to scientists trained and working in the field of bioinformatics, but with some integration of transcriptomics and ribosome profiling data, as well as an effort to make the results accessible to scientists not trained in bioinformatics, this manuscript may be relevant and of interest to researchers working on the biology of long non-coding RNAs and translation in general.

My expertise: systems biology of RNA binding proteins, transcriptomics, RNA biology.

Referee Cross-commenting

I fully agree with my co-reviewer regarding additional analyses to strengthen the manuscript.

Full Revision

Manuscript number: RC-2021-00758
Corresponding author(s): Yusuke, Suenaga

1. General Statements [optional]

The comments of the reviewers were highly insightful and enabled us to greatly improve the quality of our manuscript. We provided point-by-point responses to each of the reviewers' comments. Revisions in the text are highlighted in yellow. We hope that the revisions in the manuscript and our accompanying responses will be sufficient to make our manuscript suitable for publication.

2. Point-by-point description of the revisions

Reviewer #1

<Comment 1>
- The authors provide no rationale for using the PTI score to measure the protein-coding potential of transcripts. The only attempt to justify this measure is given in the methods: "The definition of PTI score is motivated by our hypothetical concept that translation of pPTI is limited by alternate competing sPTIs." (lines 426-427, page 20). What the PTI score measures is the dominance of the largest predicted ORF over the predicted ORFs, in terms of length. It is not clear why there would be competition for translation of putative ORFs for genuine protein-coding transcripts. An alternative hypothesis, briefly touched upon in the discussion (lines 318-320) is that translation of non-functional ORFs could give rise to the production of toxic proteins, in addition to being costly in terms of energy. The authors should provide the reasoning behind the PTI score and should explain the biological mechanisms that may underlie differences between coding and non-coding transcripts.

<Response>
Thank you for your comment. We previously identified a *de novo* gene, *NCYM*, and showed that its protein has a biochemical function (Suenaga et al 2014; Suenaga et al 2020). However, *NCYM* was previously registered as a non-coding RNA in the public database, and the established predictors for protein-coding potential, coding potential assessment tool (CPAT), showed a coding probability of NCYM of 0.022, labeling it as a noncoding RNA (new Supplementary Figure 1B). Therefore, we sought to identify a new indicator for coding potential, comparing *NCYM* with a small subset of coding and non-coding RNAs to determine whether NCYM has sequence features that would allow it to be registered as a coding transcript (data not shown). We found that predicted ORFs, other than major ORFs, seem to

be short in coding RNAs. In addition, it has been reported that upstream ORFs inhibit the translation of major ORFs (Calvo et al 2009). Therefore, we hypothesized that the predicted ORFs may reduce the translation of major ORFs, thereby becoming short in the coding transcripts, including *NCYM,* during evolution. The term ORF refers to an RNA sequence that is translated into an actual product; however, the biological significance of non-translating, predicted ORFs has been largely ignored and remains to be characterized. Therefore, we defined a PTI as an RNA sequence from the start codon sequence to the end codon sequence and did not assume that it would result in a translated product. Thus, PTI can be defined even in genuine non-coding RNAs. The major ORFs are often the longest PTIs (hereafter, primary PTIs or pPTIs) in coding transcripts. Thus, to investigate the importance of pPTIs relative to other PTIs (hereafter, secondary PTIs, or sPTIs) for the evolution of coding genes, we defined a PTI score as the occupancy of the pPTI length to the total PTI length (Figure 1A-B) and assumed that the PTI score was high in coding transcripts. These are the rationale for using the PTI score for protein-coding potential and are now included in the revised manuscript (lines 92-115, page 5-6).

To examine the biological mechanism underlying the difference between coding and noncoding RNAs, we investigated the relationship between translation and PTI scores. We chose a dataset of non-coding RNAs that translated small proteins derived from the databases SmProt and sORF.org. From ribosome profiling and mass spectrometry data, the databases include noncoding RNAs that encode small proteins (less than 100 residues) as well as mRNAs that have extra-small ORFs in addition to major ORFs. The SmProt database divides these small ORFs into three categories: upstream (uORF), small (sORF), and downstream (dORF). The definitions are based on their locations: uORFs and dORFs are located in 5' and 3' UTRs, respectively, and sORFs overlap with major ORFs using different reading frames (new Figure 2B). We first calculated PTI scores of lincRNAs encoding small proteins and found that the distribution of these lincRNAs shifted to higher PTI scores compared with the distribution of all lincRNAs (new Figure 2A). Therefore, lincRNA translation is correlated with higher PTI scores. Next, we examined whether PTI scores were associated with the translation occupancy of major ORFs in coding RNAs. We calculated PTI scores in mRNAs with uORF, sORF, or dORFs and found that the distribution of mRNAs encoding such small proteins shifted to lower PTI scores (new Figure 2C). Similar data were obtained from the sORF org dataset (Supplementary Figure 5). These data support the idea that the PTI score is related to the occupancy of the major ORF during translation. These results are now included in the results of the revised manuscript (lines 241-271, pp 12-13).

Translation of small proteins from noncoding RNAs seems to inhibit noncoding functions because of ribosome binding and subsequent translation. On the other hand, translation of sPTIs in coding RNAs seems to inhibit the translation of major ORFs because of competing translations (Calvo et al 2009). At the same time, however, the translation of such proteins

may have the advantage of producing new functional proteins/regulatory mechanisms during evolution. Therefore, the right and left shifts of the PTI score that we observed for noncoding and coding RNAs, respectively, seem to be slightly deleterious or beneficial. As further discussed in the responses below, the overlap of distributions of PTI scores between coding and noncoding transcripts was negatively correlated with the effective population size of the species. Therefore, as nearly neutral theory predicts, mutations causing such slightly deleterious/beneficial effects of translation in coding and noncoding transcripts seem to be fixed in species with small effective population sizes (including humans) by genetic drift (Kimura 1968, 1983; Ohta 1992). Clearly, PTI scores are related to translation of PTIs, and their distributions suggest a mechanism for producing bifunctional RNAs that are simultaneously coding and noncoding. The discussion has now been included in the revised manuscript (lines 487–503, pp 23–24).

Calvo SE, Pagliarini DJ, Mootha VK. Upstream open reading frames cause widespread reduction in protein expression and are polymorphic among humans. Proc Natl Acad Sci U S A. 2009 May 5;106(18):7507-12. doi: 10.1073/pnas.0810916106. Epub 2009 Apr 16. PMID: 19372376; PMCID: PMC2669787.
Kimura M. 1968. Evolutionary rate at the molecular level *Nature*. 217(5129):624-6. PMID: 5637732. https://doi.org/10.1038/217624a0
Kimura, M. (1983). *Neutral Theory of Molecular Evolution* Cambridge: Cambridge University Press. https://doi.org/10.1093/obo/9780199941728-0132
Ohta T. 1992. The Nearly Neutral Theory of Molecular Evolution. *Annu Rev Ecol Syst.* 23:263-86.

<Comment 2>
- The presence of ORFs in transcripts has long been used as a predictor of their protein-coding potential. For example, the ORF size and the ORF coverage are part of the set of predictors implemented in CPAT (Wang et al., 2013). The PTI score is necessarily related to these methods, yet no comparison is provided. If the PTI score is to be used as a measure to classify transcripts as coding or non-coding, its performance should be compared to other classifiers, including those that use the presence of ORFs as a predictor (e.g., CPAT) but not only (e.g., PhyloCSF, based on the pattern of sequence evolution).
<Response>
Thank you for your comment. As you noted, our reasons for using the PTI score were not clearly described in the original manuscript and are now included in the Results section (lines 92-115, page 5-6). As mentioned in response to comment 1, CPAT was not able to predict NCYM as a coding transcript (Supplementary Figure 1B). Furthermore, we intended to use this new concept to identify the RNA sequence elements that determine protein-coding potential, but did not intend to use the score as a classifier of coding or non-coding RNAs.

Many studies have identified bifunctional RNAs that are simultaneously coding and noncoding (Li and Liu 2019; Huang Y et al. 2021). Moreover, neutrally evolving peptides are encoded by small ORFs of noncoding RNAs, possibly contributing to the evolutionary origin of new functional proteins (Ruiz-Orera et al. 2014). Therefore, we argue that such dichotomous classification is often misleading, by unconsciously ignoring ncRNAs that encode functional or nonfunctional small proteins. Additionally, this approach has several technical problems. For a training set for use with such a classification, we need a dataset of genuine noncoding RNAs. However, it is quite difficult to define such noncoding RNAs without bias, for example, for cell or tissue types, including cancer or normal cells/tissues. Increasing evidence has shown peptide translation from known noncoding RNAs (Li and Liu 2019; Huang Y et al. 2021); moreover, some of these peptides are specific to the cellular context (Dohka et al 2021). Therefore, we cannot be certain that we are identifying genuine noncoding RNAs from the datasets from ribosome profiling or mass spectrometry, which neither cover all cell/tissue types nor all physiological contexts.

We agree with you in that we need to compare PTI scores with other indicators of coding potential, such as transcript length, ORF size, and ORF coverage. ORFs of less than 100 residues have been used to define noncoding RNAs; thus, such RNAs necessarily have shorter ORF sizes relative to coding RNAs. Therefore, we calculated these indicators by focusing on noncoding RNAs that encode proteins, but not coding RNAs (new Supplementary Figure 4). The PTI score distribution shifted to the right for lincRNAs encoding small proteins, indicating that the PTI score is related to translation (new Figure 2C). In contrast, the distributions of transcript length, ORF size, and ORF coverage did not shift higher for noncoding RNAs encoding small proteins (new Supplementary Figure 4), although a slight shift to higher ORF coverage was found. Therefore, we argue that the PTI score is a better indicator of translation than transcript length, ORF size or ORF coverage. These results are now included in the results of the revised manuscript (lines 241-255, page 12).

<Comment 3>
- The authors compare the observed PTI score distributions with the PTI scores from random or shuffled sequences. They conclude that the PTI scores do not depend on transcript lengths but on transcript sequences (lines 122-123). However, this is not true for non-coding RNAs, for which the observed and randomized distributions are very similar. The relationship between transcript length and PTI scores should be analyzed into more detail. Are the annotated non-coding transcripts with high PTI scores particular in terms of length?
<Response>
We analyzed the length of high-PTI-score transcripts compared to all lncRNA transcripts. The average high-PTI-score with high coding potential ($0.6 \leq \mathrm{PTI\ score} < 0.8$) was significantly shorter than that of all the annotated lncRNAs ($P < 7 \times 10^{-29}$), consistent with the distribution of transcript length in lincRNAs translating small proteins (new

Supplementary Figure 4C). Therefore, the high PTI scores are not simply due to the larger ORF size derived from longer transcript length, but also because of the occupancy of pPTI among all PTIs. The occupancy of pPTI can be estimated by ORF coverage or PTI score, and we can easily see that transcript length (the denominator of ORF coverage) correlates with the sum of the lengths of all PTIs (the denominator of the PTI score). Thus, we need to clarify which indicators have more biological significance in terms of gene evolution. Higher PTI scores in noncoding RNAs cause overlap of the coding and noncoding transcripts in eukaryotes, especially in multicellular eukaryotes (new Figure 4 and 5). The overlaps of PTI score distributions between coding and noncoding RNAs (O_{pti}) were positively and negatively correlated with mutation rate and effective population size, respectively, and approximated by logarithmic or exponential relationships (new Figure 6). Because the inverse of the effective population size defines the strength of genetic drift relative to the strength of selection, the overlaps quantified by O_{pti} seem to be derived from genetic drift. These results clearly suggest that the observed PTI score distribution of noncoding RNAs is not random. In contrast, ORF coverage (O_{cov}) showed a weaker relationship with mutation rates and effective population sizes (new Supplementary Figure 8 and 9). These results suggest that ORF coverage is less related to gene evolution than PTI score, with the weak relationship seemingly indirectly derived from the correlation with the PTI score. We have now included these results in the revised manuscript (lines 306-322, page 15).

<Comment 4>
- The authors discuss in depth the correlation between PTI scores and PTI-based protein-coding potential measures (e.g., section "PTI scores correlate with protein coding potential in humans and mice", starting line 125; section "Relationship between the PTI score and protein-coding potential", starting line 243). Given that the protein-coding potential is directly derived from the PTI score distributions for coding and non-coding transcripts, it is not surprising that the two should be correlated. The significance of observing a linear or a sigmoid relationship is not clearly explained.

<Response>
As you noted, the protein-coding potential was directly derived from the PTI score distributions. Therefore, if the distribution for coding RNA shows a higher or lower PTI score compared to that of noncoding RNA, the protein-coding potential is expected to be positively or negatively correlated with the PTI score. If the distributions of coding and noncoding RNA significantly overlapped ($O_{pti} > 0.7$), the protein-coding potential became constant and was not correlated with the PTI score (new Figure 7 and new Supplementary Figure 10). Thus, the PTI score is not always positively correlated with the protein-coding potential.

We had divided the species into three groups; the sigmoidal group, the linear group, or others based on the intercept and slope in the linear approximation, but considering the fit of the

Review COMMONS

linear approximation, there is no essential difference between the sigmoidal and linear groups. Therefore, in the revised text, we classify the species into two groups: linear and constant (new Figure 7 and Supplementary Figure 10). We have now replaced the figures and added a new interpretation of the results in the revised manuscript (lines 341–353, pages 16–17).

<Comment 5>

- The authors use the entire set of annotated coding and non-coding transcripts to assess the distribution of PTI scores and to define the protein-coding potential. Traditionally, for methods that aim to classify transcripts as coding or non-coding, this is done using "bona fide" coding and non-coding transcripts, which are used as training sets. The efficiency of the method can then be evaluated using a test set of transcripts. This aspect is lacking here and should be implemented.

<Response>

As we wrote in response to your comment 2, we aimed to examine what RNA sequence elements determine genuine-coding RNA but not to identify the classifier of coding and noncoding RNA. Technically, the "bona fide" coding and noncoding RNAs cannot be rigorously defined, given the possible existence of unidentified bifunctional RNAs in the testing sets; therefore, more traditional approaches often eliminate such possibilities.

<Comment 6>

- The comparisons among species are likely biased by the quality of lncRNA annotations in non-model organisms - cf. high variations among primates, which are likely driven by the annotation quality and depth.

<Response>

As written in the response to comment 3, the variation of PTI score distribution in lncRNA is not random, and overlaps with the distribution of coding RNA are negatively correlated with effective population size (new Figure 6). In addition, we found that the tissue-specific expression of lncRNA influences the PTI score distribution in multicellular eukaryotes (new Figure 8 C and D and new Supplementary Figure 11 and 12). Therefore, the variation is caused, at least in part, by the specificity of gene expression, and it thus contains biological significance. These results are now included in the revised manuscript (lines 383–402, pages 18–19).

Based on these results, we expect that the quality of the lncRNA annotations derived from two major databases, Ensenbl and RefSeq, are well curated and sufficient to compare the PTI score distribution. Realistically, there is no database that catalogs a number of curated lncRNAs from various species other than these two. However, we also expect that recent progress in whole genome sequencing and transcriptome analysis of vertebrates may improve

the annotation of lncRNAs, including non-model organisms, and provide more ideal datasets for comparisons among species.

<Comment 7>
- The differences among bacteria, archaea and eukaryotes should be discussed into more depth. In bacteria, the genuine ORF is well defined by the presence of translation signals (e.g., Shine-Dalgarno sequence). Other factors are also at work in both prokaryotes and eukaryotes, including RNA secondary structures. The relationship between these factors and the PTI score should be discussed.
<Response>
The Shine-Dalgarno sequence in bacteria and the Kozak sequence in eukaryotes have been identified as important regulatory elements for ribosome binding, but these sequences are not essential for all coding RNAs, and their significance is not well characterized, especially in noncoding RNAs that are translated. Recent research has sought to identify the determinants that regulate ribosome binding to lncRNAs using 99 characteristics, including the weight of each base at the −6 to +1 positions relative to the start codon (Kozak-like sequence) or RNA secondary structure (Zeng et al 2018). They found that transcript length is a stronger indicator than either of these characteristics for ribosome binding in human lncRNAs. Because the PTI score is a better indicator for translation of lincRNAs than transcript length (new Supplementary Figure 4C), we would argue that Kozak sequences and RNA secondary structures are not reliable indicators for ribosome binding of lncRNAs, and their significance should be limited to more specific transcript classes. Furthermore, Hata et al. recently showed that the Kozak sequence is a negative regulator of *de novo* gene birth in plants (Hata et al. 2021). Therefore, these sequence characteristics seem to evolve after the birth of coding transcripts and are not generally involved in new coding gene origination from noncoding RNAs.

Zeng C, Hamada M. 2018. Identifying sequence features that drive ribosomal association for lncRNAs *BMC Genomics*. 19(Suppl 10):906. PMID: 30598103; PMCID: PMC6311901. https://doi.org/10.1186/s12864-018-5275-8
Hata T, Satoh S, Takada N, Matsuo M, Obokata J. 2021. Kozak sequence acts as a negative regulator of de novo transcription initiation of newborn coding sequences in the plant genome. *Mol Biol Evol*. 38:2791-2803. PMID: 33705557; PMCID: PMC8233501. https://doi.org/10.1093/molbev/msab069

<Comment 8>
- From an evolutionary perspective, the effective population size (Ne) is also likely related to the "quality" of the ORFs. An analysis of Ne vs. the PTI score distributions would be an interesting addition to this manuscript.
<Response>

We appreciate this comment. We now include an analysis of the relationship between N_e and PTI scores by defining an indicator of the extent of overlap in the PTI score distributions between coding and noncoding transcripts. This overlapping score was calculated based on PTI scores or ORF coverage and named O_{pti} or O_{cov}, respectively. O_{pti} showed positive and negative correlations with mutation rates (U_p) and effective population size (N_e), respectively (new Figure 6A), suggesting that the overlap of PTI score distribution is related to slightly deleterious or beneficial mutations fixed in populations due to genetic drift. Furthermore, using the relationship between N_e and O_{pti}, we calculated the minimum effective population size to be approximately 1000, which is consistent with the results from conservation biology (Frankham et al. 2014). Indeed, species at risk of extinction had significantly higher O_{pti} than species with little risk of extinction (left panel, new Figure 6B). In addition, O_{pti} was higher for species with a decrease compared to those with stable population sizes (right panel, new Figure 6B). These results are now included in the revised manuscript (lines 323–332, page 15–16).

Frankham R, Bradshaw CJA. 2014. Genetics in conservation management: Revised recommendations for the 50/500 rules, Red List criteria and population viability analyses, Biological Conservation, 170:56–63, https://doi.org/10.1016/j.biocon.2013.12.036

<Comment 9>
Reviewer #1 (Significance (Required)):
This manuscript is lacking in novelty and is not well positioned in the field. If the aim of this work is to provide a method to classify transcripts as coding or noncoding, the authors should provide detailed comparisons with existing methods (see above). If the aim is to understand what defines a genuine protein-coding transcript, then the biological mechanisms should be better described and the comparisons among species and among functional categories of genes should be further developed. The idea of using the "dominance" of the largest ORF compared to the other predicted ORFs is interesting, and provides a new element compared to existing methods that rely exclusively on ORF length and ORF coverage. I would recommend that the authors develop this idea further and discuss the advantages of using the ORF dominance compared to just the ORF length or coverage.
<Response>
Thank you for your comment. To address this, we have revised the description of our aim to investigate what defines a genuine protein-coding transcript and found that doing so prompted us to learn that the extent of overlap of PTI score distribution between coding and noncoding transcripts is negatively correlated with effective population size. In addition, we have added characterizations of functional categories of high-PTI-score lncRNAs in mice (new Supplementary Tables 6 to 8) and *C. elegans* (new Supplementary Tables 9, 10, and 11).

Comparison of ORF size and coverage to PTI score showed that PTI score is a better indicator for translation of lncRNAs than these indicators and has biological significance in molecular evolution because of the clear correlation between mutation rate and effective population size. These results and related descriptions are now included in the revised manuscript (lines 323–332, pages 15–16; lines 210–218, pages 10–11).

<Comment 10>
****Referee Cross-commenting****
I fully agree with Reviewer 2's remarks. In particular, adding ribosome profiling analyses is an excellent idea and could substantially improve the manuscript.
<Response>
We investigated the PTI scores in lncRNAs that are translated, using ribosome profiling data, and found that PTI scores correlated with translation (lines 241–271, pages 12–13). Thank you for this excellent suggestion.

Reviewer 2

****Major comments:****
<Comment 1>
- some validation of their predictions of coding potential would be good to add. There are plenty of ribosome profiling experiments out there for some of the studied organisms (human, mouse, E. coli) that could be used to show that indeed some of the non-coding RNAs are misclassified and have ribosome density across the predicted open reading frames.
<Response>
Thank you for your comment. As noted in our response to Reviewer 1 above, we calculated the PTI scores of translated lncRNAs from the two databases and found that the PTI score correlates with translation of both coding and noncoding RNAs (new Figure 2 and new Supplementary Figures 4 and 5). As noted above, such translation seems to produce slightly deleterious/beneficial effects, thereby becoming fixed in species with smaller effective population sizes by genetic drift. These results and related discussion are now included in the revised manuscript (lines 241–271, pages 12–13; lines 323–332, pages 15–16; lines 487–503, page 23–24).

<Comment 2>
- the manuscript is at times difficult to follow and the implication of the statements may not be immediately clear to the readers, particularly those without formal training in bioinformatic methods; even in the abstract. Some examples: "The relationship between the PTI score and protein-coding potential was sigmoidal in most eukaryotes; however, it was linear passing through the origin in three distinct eutherian lineages, including humans". Here

it is not clear what this means (without reading the paper) – and even after reading the paper the importance of noting the sigmoidal vs linear relationship of PTI vs. protein-coding potential is unclear. I would encourage the authors to double-check that they provide a clear interpretation of their results, with readers unschooled in proper statistics in mind.
<Response>
Thank you for these comments. As we noted in response to comment 4 of Reviewer 1, considering the fit of the linear approximation, there was no essential difference between the sigmoidal and linear groups. Therefore, in the revised manuscript, we classify the species into two groups: linear and constant (new Figure 7 and Supplementary Figure 10). We also propose and diagram a new gene birth model to help readers understand our interpretations more easily (Figure 9). These results and discussion are now included in the revised manuscript (lines 341-353, pages 16-17; lines 514-538, pages 24-25).

<Comment 3>
- For the definition of PTI and protein-coding potential the authors refer to the Materials and Methods. I would encourage to explain in plain terms in the results section 1.) how they decided on this particular formalization and 2.) explain clearly what this means.
<Response>
Thank you for your suggestion. We have included a concise definition in the revised text in plain terms (lines 107-115, page 5-6; lines 144-146, page 7).

<Comment 4>
- The definition of protein coding potential for appears to be dependent on database classification of a transcript as either coding and non-coding. Particularly for organisms with complex transcriptomes, databases may not contain the proper information - what are the implications for their protein-coding potential score?
<Response>
Organisms with complex transcriptomes, such as multicellular organisms, present difficulties in classifying coding vs. noncoding transcripts because RNAs classified as noncoding based on proteomic data from a subset of cell types may encode functional proteins in other cell types for which proteomic data are not available. To examine whether cell types affect the PTI distribution of coding and noncoding transcripts, we analyzed transcriptomic data from five mammals (human, mouse, rat, macaque, and opossum) and found that the PTI score distributions were similar in most cell or tissue types for noncoding transcripts (new Figure 8C and Supplementary Figure 11). However, PTI score distributions for noncoding RNA in mature testes showed a rightward shift for all five species (new Figure 8C and Supplementary Figure 11).

Furthermore, we found that tissue specificity of RNA expression was correlated with PTI score (new Figure 8D and new Supplementary Figure 12 and 13), with more specific

expression associated with higher PTI scores in all five species, with the majority of the tissue-specific expression in mature testis. Therefore, the mature testis is a special tissue that expresses noncoding RNAs with high coding potentials. These results support the hypothesis that the testis is a special organ for new gene origination (Kaessmann 2010). We have added these results and discussion to the revised manuscript (lines 383–402, pages 18–19; lines 427–434, pages 20–21; lines 435–445, page 21).

Kaessmann H. 2010. Origins, evolution, and phenotypic impact of new genes. Genome Res, 20:1313–26. Epub 2010 Jul 22. PMID: 20651121; PMCID: PMC2945180. https://doi.org/10.1101/gr.101386.109

<Comment 5>
– The authors completely ignore plants – would it make sense to expand their analysis to this branch of the tree of life?
<Response>
In Supplementary Figure 5 of our original manuscript (new Supplementary Figure 7), we have included the PTI score distributions from plants. We also present their overlapping scores (O_{pti}) in the revised manuscript.

<Comment 6>
Reviewer 2 (Significance (Required)):
The manuscript presents an elegant way to predict protein-coding and non-coding RNAs, which may be very relevant to the study of organisms with complex transcriptomes. The audience for the manuscript at the moment may be more limited to scientists trained and working in the field of bioinformatics, but with some integration of transcriptomics and ribosome profiling data, as well as an effort to make the results accessible to scientists not trained in bioinformatics, this manuscript may be relevant and of interest to researchers working on the biology of long non-coding RNAs and translation in general. My expertise: systems biology of RNA binding proteins, transcriptomics, RNA biology.

****Referee Cross-commenting****
I fully agree with my co-reviewer regarding additional analyses to strengthen the manuscript.

<Response>
Thank you for these comments. We analyzed noncoding RNAs using ribosome profiling data and transcriptomes in different tissues. We found that high PTI scores correlated with translation of noncoding RNAs, and that such high PTI-score noncoding RNAs were specifically expressed in mature testes. Because the effective population size was inversely correlated with the overlap of PTI distributions, the slightly deleterious or beneficial mutations in germ cells of matured testis seem to generate high-PTI score noncoding RNAs

as candidates for new coding genes in the next generation. This idea is consistent with the hypothesis that new coding transcripts are derived from noncoding transcripts expressed in spermatocytes and spermatids in mature testes. In addition, we found that human noncoding transcripts with high PTI scores tended to be involved in transcriptional regulation, and the target gene of MYCN was significantly enriched as the original gene. A recent study showed that binding sites for transcription factors, including MYCN, are mutational hotspots in human spermatogonia (Kaiser et al. 2021). Therefore, the PTI score offers an opportunity to integrate the concept of gene birth with classical molecular evolutionary theory, thereby contributing to our understanding of evolution.

Kaiser VB et al. 2021. Mutational bias in spermatogonia impacts the anatomy of regulatory sites in the human genome. *Genome Res*. Epub ahead of print. PMID: 34417209. https://doi.org/10.1101/gr.275407.121

16th Dec 2021
Dear Dr. Suenaga,

Thank you for your patience while your revised manuscript was peer-reviewed at EMBO reports. We have now received the comments from both referees and I discussed your study and the reports also with my colleagues here, including our chief editor Bernd Pulverer.

As you will see, referee 1 and 2 are not in agreement. While referee 1 is more positive, referee 2 raises important points, and based on these we have decided that we cannot offer to publish your manuscript as it stands now. EMBO reports only allows one major round of revision, and addressing all controls referee 2 suggests and that would be required to strengthen the study, will entail another round of major revisions.

I am sorry that this decision emerges as the outcome of a lengthy revision process, but we agree with the comments by referee 2 that the study is not sufficiently strong as it stands now.

Having said that, if you think that you can successfully address referee 2's comments, we would be willing to consider a significantly strengthened study on the topic in the future. Please note that the novelty of the findings will be re-assessed in case of re-submission of a significantly revised study.

While we cannot pursue this manuscript further at EMBO report as it stands now, we would like to notify you of the possibility to transfer your study to our not-for-profit open-access sister journal, Life Science Alliance (LSA). We shared your manuscript and the accompanying reviews with LSA Executive Editor, Eric Sawey, who is interested in these findings, and would like to publish this manuscript at LSA pending the following revisions: - Address Reviewer 1's request for clarity - Address Reviewer 2's suggestion to rename PTI "Orf dominance", address the comments about "noncoding RNA that encode proteins", discuss the potential implications of excluding very short ORFs, and address the comments made about the Ne analysis and testis expression. We encourage you to use the link below to transfer your manuscript to LSA. You do not need to revise the manuscript before transferring it to LSA. Once you transfer, Dr. Sawey will email you an invitation to revise and resubmit, listing the same revision requests as mentioned above. Please feel free to reach out at e.sawey@life-science-alliance.org if you have any questions about the LSA journal, the transfer process or the revisions requested.

I hope that you will be interested in one of the two options we can offer to proceed with your study and its timely publication at EMBO press.

Kind regards,
Esther

Esther Schnapp, PhD
Senior Editor
EMBO reports

Referee #1:

The authors comprehensively addressed the reviewers' concerns. In my mind the conclusions of the manuscript are well-supported by their analyses and the manuscript is suitable for EMBO Reports and could be a useful resource for researchers interested in understanding coding potential of non-coding RNAs.
That being said, I highly encourage the authors to shorten the manuscript and edit for clarity, in particular sections that will be referenced by most readers, Abstract and Discussion. I found the new Abstract better suited for general audiences, nevertheless, it is difficult to follow and several points raised in the Abstract only become clear after reading the Discussion (e.g. population size, how multicellularity gives rise to bifunctional transcripts, the role of the testes). Please consider careful editing for clarity and for better accessibility for a broader audience!

Referee #2:

In the reply to reviewers, the authors say that "the term ORF refers to an RNA sequence that is translated into an actual product". That is not by no means the accepted definition. Open reading frames are usually defined as sequences that do not contain stop codons (that may or may not start with a start codon), which could in principle be translated - but evidence of translation is generally not available and thus not required. Putative translation islands, as defined here, are thus indistinct from ORFs. In my original review, I recommended that the PTI score should be renamed to something along the lines of "ORF dominance". I was perhaps not clear enough: I see no reason to introduce the "putative translated island" notion, given that it is entirely redundant with the ORF notion. I again recommend that this notion be reformulated for clarity.

Terminology put aside, I am open to the notion of "ORF dominance" as a useful measure for identifying RNAs that can give rise to functional proteins, but the manuscript has still not brought sufficient evidence for this beyond was already known about the presence of upstream ORFs in mRNAs. The analyses presented in the manuscript often lack the proper controls. In both initial reviews, it was recommended that the authors use ribosome profiling data to test their hypotheses regarding the efficiency of translation for transcripts with high PTI scores. In the revised version, the authors do this only by analyzing databases of lncRNAs for which ribosome profiling data suggests that there are small encoded proteins. They find that PTI scores of these lncRNAs are higher, but this is obvious given that these transcripts include open reading frames large enough to be detected as translated with ribosome profiling data. What about lncRNAs that have similar sized ORFs, but without evidence of translation from ribosome profiling?
Also, what about protein-coding mRNAs with low PTI scores? Are they less efficiently translated? The authors say that "mRNAs with uORFs, sORFs, dORFs" have PTI scores that shift to lower levels. That is obvious, given the definition of the PTI score. Clearly if other ORFs can be identified, the ORF dominance score will be lower. Here, a proper control would be to compare mRNAs with small, *translated* open reading frames with mRNAs that have small, *untranslated* ORFs. Analysis of ribosome profiling data in conjunction with RNA-seq should also give an idea of translation efficiency. The authors could use for example data from Wang et al, Transcriptome and translatome co-evolution in mammals, Nature, 2020.

The authors use throughout the paper the notion of "noncoding RNA that encode proteins". It would be better to classify transcripts as (potentially) coding, non-coding and bifunctional. The authors should keep in mind that for the vast majority of lncRNAs that are annotated in classical databases there is absolutely no evidence that they function as RNA molecules. They were defined as non-coding RNAs based on the absence of evidence that they code for proteins. If with additional experiments (as for the authors' own NCYM example) it turns out that these lncRNAs indeed encode small peptides, the logical step would be to reclassify them as coding transcripts, given that we still have no evidence that they function as RNAs.

Regarding my comment on the relationship between high PTI scores and transcript length for ncRNAs, the authors show that the ncRNAs with high PTI scores are shorter than the average of all ncRNAs. This was indeed my expectation. In longer transcripts, more ORFs can be identified just by chance, while in shorter transcripts, the likelihood of observing ORFs is smaller, and the likelihood of observing more than one ORF is very low - thus the PTI scores are larger simply due to the short transcript length. I noticed also that the authors exclude transcripts that have very short ORFs, shorter than 20 amino-acids (lines 122-124). This is likely to bias the results.

Regarding the Ne analysis: the nearly neutral theory does not predict that slightly beneficial mutations would get fixed easier in populations with small Ne compared to populations with large Ne! This is only true for slightly deleterious mutations. On the contrary, slightly beneficial mutations are more likely to get eliminated in populations with small Ne than in populations with large Ne.

Regarding the testis expression: please also look into Soumillon et al, Cell Reports, 2013. It is not just the case that the testis expresses more "bifunctional" transcripts, in fact it expresses *everything*, including many nonfunctional transcripts.

Response to Referee 1

<Comment 1>

The authors comprehensively addressed the referees' concerns. In my mind the conclusions of the manuscript are well-supported by their analyses and the manuscript is suitable for EMBO Reports and could be a useful resource for researchers interested in understanding coding potential of non-coding RNAs.

That being said, I highly encourage the authors to shorten the manuscript and edit for clarity, in particular sections that will be referenced by most readers, Abstract and Discussion. I found the new Abstract better suited for general audiences, nevertheless, it is difficult to follow and several points raised in the Abstract only become clear after reading the Discussion (e.g. population size, how multicellularity gives rise to bifunctional transcripts, the role of the testes). Please consider careful editing for clarity and for better accessibility for a broader audience!

<Response>

We thank Referee 1 for the kind comments and suggestions. The paper has been carefully revised to improve text consistency and clarity. In the Abstract, we tried to avoid the use of conceptual expressions, instead using more results-oriented expressions to facilitate readers' understanding or our findings and conclusions (page 2, lines 34-37). In the Discussion section, detailed descriptions of *G. lamblia*, feedback regulation of NCYM in cancer, and general descriptions of viruses in relation to vORF scores were removed because they fall outside the main focus of this paper. Description and discussions related to beneficial and deleterious mutations were also deleted, as it is better to wait for the results of future population genetic and molecular biological analyses to further the claims previously made.

Responses to Referee 2

<Comment 1>

In the reply to referees, the authors say that "the term ORF refers to an RNA sequence that is translated into an actual product". That is not by no means the accepted definition. Open reading frames are usually defined as sequences that do not contain stop codons (that may or may not start with a start codon), which could in principle be translated - but evidence of translation is generally not available and thus not required. Putative translation islands, as defined here, are thus indistinct from ORFs. In my original review, I recommended that the PTI score should be renamed to something along the lines of "ORF dominance". I was perhaps

not clear enough: I see no reason to introduce the "putative translated island" notion, given that it is entirely redundant with the ORF notion. I again recommend that this notion be reformulated for clarity.

<Response>
We thank Referee 2 for this comment. We agree that the concept of PTI overlaps with that of ORF; it was only introduced to clarify which sequences were used to calculate the ORF dominance score. We believe that ORF dominance calculation is now clearly mentioned in the materials and methods section. We deleted the terms PTI and PTI score from the revised manuscript and use ORF and ORF dominance throughout the revised manuscript.

<Comment 2>
Terminology put aside, I am open to the notion of "ORF dominance" as a useful measure for identifying RNAs that can give rise to functional proteins, but the manuscript has still not brought sufficient evidence for this beyond was already known about the presence of upstream ORFs in mRNAs. The analyses presented in the manuscript often lack the proper controls. In both initial reviews, it was recommended that the authors use ribosome profiling data to test their hypotheses regarding the efficiency of translation for transcripts with high PTI scores. In the revised version, the authors do this only by analyzing databases of lncRNAs for which ribosome profiling data suggests that there are small encoded proteins. They find that PTI scores of these lncRNAs are higher, but this is obvious given that these transcripts include open reading frames large enough to be detected as translated with ribosome profiling data. What about lncRNAs that have similar sized ORFs, but without evidence of translation from ribosome profiling?

<Response>
As shown in the new Figure 2A (bottom left), the length of the primary ORF, that is, ORF size, did not change between small protein-encoding lincRNAs and lincRNAs without evidence of translation. Nevertheless, ORF dominance shifted to higher values for lincRNAs with translation (top left, new Figure 2A), whereas ORF coverage remained almost unchanged (top center, new Figure 2A). The shift of ORF dominance to higher values is due to the relatively shorter length of lincRNA transcripts with translation (top right, new Figure 2A) and to the lower sum of secondary ORF lengths, as Referee 2 points out in comment 5 (see below). We thank Referee 2 for this valuable insight. The Results and Discussion sections of the revised manuscript have been updated to include the information stated above (lines 235-251, pages 11-12).

<Comment 3>

Also, what about protein-coding mRNAs with low PTI scores? Are they less efficiently translated? The authors say that "mRNAs with uORFs, sORFs, dORFs" have PTI scores that shift to lower levels. That is obvious, given the definition of the PTI score. Clearly if other ORFs can be identified, the ORF dominance score will be lower. Here, a proper control would be to compare mRNAs with small, *translated* open reading frames with mRNAs that have small, *untranslated* ORFs. Analysis of ribosome profiling data in conjunction with RNA-seq should also give an idea of translation efficiency. The authors could use for example data from Wang et al, Transcriptome and translatome co-evolution in mammals, Nature, 2020.

<Response>

We thank Referee 2 for this suggestion. The reference cited defines a dominant isoform for each gene and examines the translation efficiency of each gene. We contacted the authors and they kindly provided us the transcript ID of the dominant isoform of each gene, and we then calculated ORF dominance.

Translation of transcripts in spermatocytes and spermatids, is strongly downregulated on average. However, the authors identified a gene set (cluster I genes) that efficiently translated in the spermatocytes and spermatids, therefore escaping the overall translational repression (Wang et al. 2020). We found that cluster I genes showed higher ORF dominance than cluster III genes that showed translational repression in spermatocytes and spermatids (new Figure 2B). Furthermore, coding transcripts with translation from multiple ORFs showed significantly low or high percentages of cluster I or cluster III genes, respectively, compared with those without evidence of translation (new Figure 2C). These results support the idea that ORF dominance is associated with translation efficiency in coding transcripts. We deleted the previous Figure 2B, 2C, and Supplementary Figure 5 and added these results to the new Figure 2B and 2C and to the Results section (lines 252-262, page 12), and discuss them in the Discussion section (lines 435-442, pages 20-21).

<Comment 4>

The authors use throughout the paper the notion of "noncoding RNA that encode proteins". It would be better to classify transcripts as (potentially) coding, non-coding and bifunctional. The authors should keep in mind that for the vast majority of lncRNAs that are annotated in classical databases there is absolutely no evidence that they function as RNA

molecules. They were defined as non-coding RNAs based on the absence of evidence that they code for proteins. If with additional experiments (as for the authors' own NCYM example) it turns out that these lncRNAs indeed encode small peptides, the logical step would be to reclassify them as coding transcripts, given that we still have no evidence that they function as RNAs.

<Response>
We thank Referee 2 for these helpful suggestions. Throughout the paper, we have avoided using phrases such as "noncoding RNAs that encode proteins" instead using (potentially) bifunctional RNAs or nonfunctional RNAs for those with intermediate ORF dominance. We have deleted or replaced the following four sentences:

1) lines 412-413, page 20, previous text
We deleted "Moreover, functional noncoding RNAs, by definition, should not be translated by ribosomes in cells."

2) lines 416-417, page 20, previous text (lines 408-409, page 19, revised text)
We replaced "transcripts with noncoding functions" to "lncRNAs" in "Thus, as expected, transcripts with noncoding functions in bacteria and archaea showed low ORF dominance (Figure 9A, top panel)".

3) lines 419-420, page 20, previous text (lines 412, page 19, revised text)
We replaced "function" with "exist" in "Therefore, eukaryotic lncRNAs may exist in the nucleus even with high ORF dominance".

4) lines 422-424, page 20, previous text (lines 415, page 19, revised text)
We deleted "functional" from "Thus, the pervasive transcription of the genome seems to help eukaryotes to produce new functional noncoding/coding RNAs"

In addition, the terms "nonfunctional" and "bifunctional" were added to Figure 9B, and relevant discussion was added to the revised text (lines 430-432, page 20; lines 467-468, page 22).

<Comment 5>
Regarding my comment on the relationship between high PTI scores and transcript length for ncRNAs, the authors show that the ncRNAs with high PTI scores are shorter than the

average of all ncRNAs. This was indeed my expectation. In longer transcripts, more ORFs can be identified just by chance, while in shorter transcripts, the likelihood of observing ORFs is smaller, and the likelihood of observing more than one ORF is very low - thus the PTI scores are larger simply due to the short transcript length. I noticed also that the authors exclude transcripts that have very short ORFs, shorter than 20 amino-acids (lines 122-124). This is likely to bias the results.

<Response>
As shown in the response to Comment 2 of Referee 2, the higher ORF dominance of shorter transcripts (as assumed by the Referee) was due to the low value obtained for the sum of secORFs lengths (Figure 2A). We thank Referee 2 for this valuable insight. The exclusion of lncRNAs with pORFs less than 20 amino acids has been used in several studies on lncRNA translation (e.g., Bazzini et al. 2014; Ruiz-Orera et al. 2014; Schmitz et al. 2018), and we also excluded them because the existence or physiological significance of such short peptides is not clear in the majority of the species analyzed in the present study. We have added this information to the revised text (lines 118-119, page 6; lines 517-519, page 24).

<Comment 6>
Regarding the Ne analysis: the nearly neutral theory does not predict that slightly beneficial mutations would get fixed easier in populations with small Ne compared to populations with large Ne! This is only true for slightly deleterious mutations. On the contrary, slightly beneficial mutations are more likely to get eliminated in populations with small Ne than in populations with large Ne.

<Response>
We thank Referee 2 for pointing out this mistake. Accordingly, the description of slightly beneficial mutations and its relevant discussion (lines 488-503, pages 22-23, previous text) were deleted and “neutral/slightly deleterious mutations” are now referred in the revised text (lines 479-480, page 22).

<Comment 7>
Regarding the testis expression: please also look into Soumillon et al, Cell Reports, 2013. It is not just the case that the testis expresses more "bifunctional" transcripts, in fact it expresses *everything*, including many nonfunctional transcripts.

<Response>

We thank Referee 2 for this important comment. We agree and explicitly state in the revised manuscript that the transcripts expressed in testes include not only potentially bifunctional RNAs but also nonfunctional noncoding RNAs (Discussion section of the revised manuscript, lines 430-432, page 20).

1st Revision - Editorial Decision 18th Mar 2022

Dear Dr. Suenaga,

Thank you for the submission of your revised manuscript. We have now received the enclosed report from referee 2, and I am happy to say that this referee agrees now with the publication of your study here. We can therefore in principle accept your manuscript.

However, a few editorial requests still need to be addressed:

- The definition of the ORF dominance score could be clearer in the abstract and introduction. It could be better defined

a) that by primary ORF you mean the longest of all ORFs of a given RNA (correct?)
b) what exactly you mean by "occupancy". I think it is the fraction the primary ORF length constitutes in the sum of all putative ORFs lengths (correct?). Occupancy is normally used for ribosome occupancy, and if this is not meant here, it might be better to chose a different term. It might be useful to discuss the entire manuscript with a native speaker for a clear description of your definition and your data.

- Please add up to 5 keywords to the manuscript file.

- Please update the conflict of interest subheading to "Disclosure and competing interest statement"

- In the author contributions, there are 2 MKs; one should be MKa and the other MKo. The online Author Contributions are missing, please add these when you upload your final manuscript files.

- In the REFERENCE FORMAT the year needs to be in brackets (Harvard style).

- Please remove data not shown on page 5, as per journal policy.

- Please complete our author checklist, which you can download from our author guidelines <https://www.embopress.org/page/journal/14693178/authorguide>. The completed author checklist will also be part of our transparent review process file (RPF).

- Please upload all main and all Expanded View figures as separate files.

- The figures need to be uploaded as individual production quality figure files such as .eps, .tif, .jpg (one file per figure). See https://wol-prod-cdn.literatumonline.com/pb-assets/embo-site/EMBOPress_Figure_Guidelines_061115-1561436025777.pdf for more info on how to prepare your figures. See also the guidelines for figure legend preparation: https://www.embopress.org/page/journal/14693178/authorguide#figureformat

- We replaced Supplementary Information with Expanded View (EV) Figures and Tables that are collapsible/expandable online. A maximum of 5 EV Figures can be typeset. EV Figures should be cited as 'Figure EV1, Figure EV2" etc... in the text and their respective legends should be included in the main text after the legends of the regular figures.

- For the figures that you do NOT wish to display as Expanded View figures, they should be bundled together with their legends in a single PDF file called *Appendix*, which should start with a short Table of Content (including page numbers). Appendix figures should be referred to in the main text as: "Appendix Figure S1, Appendix Figure S2" etc. See detailed instructions regarding expanded view here: <https://www.embopress.org/page/journal/14693178/authorguide#expandedview>
You can also bundle all supplemental figures that you have in an Appendix file, if this is easier, and not chose any EV figures.

- I suggest that you upload your supplementary tables as separate Dataset files (called Dataset EV1, etc). The legends need to be provided in a separate tab in the individual excel files.

- The supplementary note needs to be deleted, and I suggest to move the information to the methods section.

- I attach to this email a related manuscript file with comments by our data editors. Please address all comments in the final manuscript file.

I would like to suggest a few changes to the title and abstract that needs to be written in present tense. Please let me know whether you agree with the following:

Protein-coding potential of RNAs measured by open reading frame dominance

or

Open reading frame dominance indicates protein-coding potential of RNAs

Recent studies have identified numerous RNAs with both coding and noncoding functions. However, the sequence characteristics that determine this bifunctionality remain largely unknown. In the present study, we develop and test the open reading frame (ORF) dominance score, which defines the occupancy of the longest ORF among all putative ORFs. This score correlates with translation efficiency in coding transcripts and with translation of noncoding RNAs. In bacteria and archaea, coding and noncoding transcripts have narrow distributions of high and low ORF dominance, respectively, whereas those of eukaryotes show relatively broader ORF dominance distributions, with considerable overlap between coding and noncoding transcripts. The extent of overlap positively and negatively correlates with the mutation rate of genomes and the effective population size of species, respectively. Tissue-specific transcripts show higher ORF dominance than ubiquitously expressed transcripts, and the majority of tissue-specific transcripts are expressed in mature testes. These data suggest that the decrease in population size and the emergence of testes in eukaryotic organisms allowed for the evolution of potentially bifunctional RNAs.

EMBO press papers are accompanied online by A) a short (1-2 sentences) summary of the findings and their significance, B) 2-3 bullet points highlighting key results and C) a synopsis image that is exactly 550 pixels wide and 200-600 pixels high (the height is variable). You can either show a model or key data in the synopsis image. Please note that text needs to be readable at the final size. Please send us this information along with the revised manuscript.

I look forward to seeing a final version of your manuscript as soon as possible. Please use this link to submit your revision: https://embor.msubmit.net/cgi-bin/main.plex

Do not hesitate to contact me should you have any questions.

Best regards,
Esther

Esther Schnapp, PhD
Senior Editor
EMBO reports

Referee #2:

I would like to thank the authors for taking into account my suggestions. In this revised version, they include analyses that clarify the relationship between transcript length and lncRNA translation potential. They have also included an additional analysis of translation efficiency, based on processed data from Wang et al., Nature 2020. Although this is not what I had in mind (the analysis they include only compares three classes of genes, of varying translation efficiency in spermatocytes and spermatids; they do not analyze data on the other tissues studied by Wang et al - brain and liver, specifically), I appreciate the effort they put into this.

I remain convinced that the ORF dominance score is a useful feature to classify putative coding and non-coding transcripts, that could be used in conjunction with other classification criteria (i.e., codon substitution frequency scores etc.). I still believe that the manuscript would be better suited for a more specialised journal but I see no reason to oppose publication in EMBO Reports.

The authors have addressed all minor editorial requests.

Dr. Yusuke Suenaga
Chiba Cancer Center Research Institute
666-2 Nitona, Chuo-ku
Chiba 260-8717
Japan

Dear Dr. Suenaga,

I am very pleased to accept your manuscript for publication in the next available issue of EMBO reports. Thank you for your contribution to our journal.

At the end of this email I include important information about how to proceed. Please ensure that you take the time to read the information and complete and return the necessary forms to allow us to publish your manuscript as quickly as possible.

As part of the EMBO publication's Transparent Editorial Process, EMBO reports publishes online a Review Process File to accompany accepted manuscripts. As you are aware, this File will be published in conjunction with your paper and will include the referee reports, your point-by-point response and all pertinent correspondence relating to the manuscript.

If you do NOT want this File to be published, please inform the editorial office within 2 days, if you have not done so already, otherwise the File will be published by default [contact: emboreports@embo.org]. If you do opt out, the Review Process File link will point to the following statement: "No Review Process File is available with this article, as the authors have chosen not to make the review process public in this case."

Should you be planning a Press Release on your article, please get in contact with emboreports@wiley.com as early as possible, in order to coordinate publication and release dates.

Thank you again for your contribution to EMBO reports and congratulations on a successful publication. Please consider us again in the future for your most exciting work.

Yours sincerely,

Esther Schnapp, PhD
Senior Editor
EMBO reports

****

THINGS TO DO NOW:

Please note that you will be contacted by Wiley Author Services to complete licensing and payment information. The required 'Page Charges Authorization Form' is available here: https://www.embopress.org/pb-assets/embo-site/er_apc.pdf

You will receive proofs by e-mail approximately 2-3 weeks after all relevant files have been sent to our Production Office; you should return your corrections within 2 days of receiving the proofs.

Please inform us if there is likely to be any difficulty in reaching you at the above address at that time. Failure to meet our deadlines may result in a delay of publication, or publication without your corrections.

All further communications concerning your paper should quote reference number EMBOR-2021-54321V3 and be addressed to emboreports@wiley.com.

Should you be planning a Press Release on your article, please get in contact with emboreports@wiley.com as early as possible, in order to coordinate publication and release dates.

EMBO Press Author Checklist

Corresponding Author Name: Yusuke Suenaga	
Journal Submitted to: EMBO Reports	
Manuscript Number: EMBOR-2021-54321V2	

USEFUL LINKS FOR COMPLETING THIS FORM
The EMBO Journal - Author Guidelines
EMBO Reports - Author Guidelines
Molecular Systems Biology - Author Guidelines
EMBO Molecular Medicine - Author Guidelines

Reporting Checklist for Life Science Articles (updated January 2022)
This checklist is adapted from Materials Design Analysis Reporting (MDAR) Checklist for Authors. MDAR establishes a minimum set of requirements in transparent reporting in the life sciences (see Statement of Task: 10.31222/osf.io/9sm4x). Please follow the journal's guidelines in preparing your manuscript.
Please note that a copy of this checklist will be published alongside your article.

Abridged guidelines for figures
1. Data
The data shown in figures should satisfy the following conditions:
- the data were obtained and processed according to the field's best practice and are presented to reflect the results of the experiments in an accurate and unbiased manner.
- ideally, figure panels should include only measurements that are directly comparable to each other and obtained with the same assay.
- plots include clearly labeled error bars for independent experiments and sample sizes. Unless justified, error bars should not be shown for technical replicates.
- if n<5, the individual data points from each experiment should be plotted. Any statistical test employed should be justified.
- Source Data should be included to report the data underlying figures according to the guidelines set out in the authorship guidelines on Data Presentation.

2. Captions
Each figure caption should contain the following information, for each panel where they are relevant:
- a specification of the experimental system investigated (eg cell line, species name).
- the assay(s) and method(s) used to carry out the reported observations and measurements.
- an explicit mention of the biological and chemical entity(ies) that are being measured.
- an explicit mention of the biological and chemical entity(ies) that are altered/varied/perturbed in a controlled manner.
- the exact sample size (n) for each experimental group/condition, given as a number, not a range;
- a description of the sample collection allowing the reader to understand whether the samples represent technical or biological replicates (including how many animals, litters, cultures, etc.).
- a statement of how many times the experiment shown was independently replicated in the laboratory.
- definitions of statistical methods and measures:
  - common tests, such as t-test (please specify whether paired vs. unpaired), simple χ2 tests, Wilcoxon and Mann-Whitney tests, can be unambiguously identified by name only, but more complex techniques should be described in the methods section;
  - are tests one-sided or two-sided?
  - are there adjustments for multiple comparisons?
  - exact statistical test results, e.g., P values = x but not P values < x;
  - definition of 'center values' as median or average;
  - definition of error bars as s.d. or s.e.m.

Please complete ALL of the questions below.
Select "Not Applicable" only when the requested information is not relevant for your study.

Materials

Newly Created Materials	Information included in the manuscript?	In which section is the information available? (Reagents and Tools Table, Materials and Methods, Figures, Data Availability Section)
New materials and reagents need to be available; do any restrictions apply?	Not Applicable	

Antibodies	Information included in the manuscript?	In which section is the information available? (Reagents and Tools Table, Materials and Methods, Figures, Data Availability Section)
For **antibodies** provide the following information:<br>- Commercial antibodies: RRID (if possible) or supplier name, catalogue number and or/clone number<br>- Non-commercial: RRID or citation	Not Applicable	

DNA and RNA sequences	Information included in the manuscript?	In which section is the information available? (Reagents and Tools Table, Materials and Methods, Figures, Data Availability Section)
**Short novel DNA or RNA including primers, probes**: provide the sequences.	Not Applicable	

Cell materials	Information included in the manuscript?	In which section is the information available? (Reagents and Tools Table, Materials and Methods, Figures, Data Availability Section)
**Cell lines:** Provide species information, strain. Provide accession number in repository **OR** supplier name, catalog number, clone number, and/**OR** RRID.	Not Applicable	
**Primary cultures:** Provide species, strain, sex of origin, genetic modification status.	Not Applicable	
Report if the cell lines were recently **authenticated** (e.g., by STR profiling) and tested for mycoplasma contamination.	Not Applicable	

Experimental animals	Information included in the manuscript?	In which section is the information available? (Reagents and Tools Table, Materials and Methods, Figures, Data Availability Section)
**Laboratory animals or Model organisms:** Provide species, strain, sex, age, genetic modification status. Provide accession number in repository **OR** supplier name, catalog number, clone number, **OR** RRID.	Not Applicable	
**Animal observed in or captured from the field:** Provide species, sex, and age where possible.	Not Applicable	
Please detail **housing and husbandry conditions**.	Not Applicable	

Plants and microbes	Information included in the manuscript?	In which section is the information available? (Reagents and Tools Table, Materials and Methods, Figures, Data Availability Section)
**Plants:** provide species and strain, ecotype and cultivar where relevant, unique accession number if available, and source (including location for collected wild specimens).	Not Applicable	
**Microbes:** provide species and strain, unique accession number if available, and source.	Not Applicable	

Human research participants	Information included in the manuscript?	In which section is the information available? (Reagents and Tools Table, Materials and Methods, Figures, Data Availability Section)
If collected and within the bounds of privacy constraints report on age, sex and gender or ethnicity for all study participants.	Not Applicable	

Core facilities	Information included in the manuscript?	In which section is the information available? (Reagents and Tools Table, Materials and Methods, Figures, Data Availability Section)
If your work benefited from core facilities, was their service mentioned in the acknowledgments section?	Not Applicable	

Design

Study protocol	Information included in the manuscript?	In which section is the information available? (Reagents and Tools Table, Materials and Methods, Figures, Data Availability Section)
If study protocol has been **pre-registered, provide DOI in the manuscript**. For clinical trials, provide the trial registration number **OR** cite DOI.	Not Applicable	
Report the **clinical trial registration number** (at ClinicalTrials.gov or equivalent), where applicable.	Not Applicable	

Laboratory protocol	Information included in the manuscript?	In which section is the information available? (Reagents and Tools Table, Materials and Methods, Figures, Data Availability Section)
Provide DOI OR other citation details if **external detailed step-by-step protocols** are available.	Not Applicable	

Experimental study design and statistics	Information included in the manuscript?	In which section is the information available? (Reagents and Tools Table, Materials and Methods, Figures, Data Availability Section)
Include a statement about **sample size** estimate even if no statistical methods were used.	Yes	Figure legends, Materials and Methods, and Expanded View Tables
Were any steps taken to minimize the effects of subjective bias when allocating animals/samples to treatment (e.g. **randomization procedure**)? If yes, have they been described?	Not Applicable	
Include a statement about **blinding** even if no blinding was done.	Not Applicable	
Describe **inclusion/exclusion criteria** if samples or animals were excluded from the analysis. Were the criteria pre-established?<br><br>If sample or data points were omitted from analysis, report if this was due to attrition or intentional exclusion and provide justification.	Yes	Figure legends and Materials and Methods
For every figure, are **statistical tests** justified as appropriate? Do the data meet the assumptions of the tests (e.g., normal distribution)? Describe any methods used to assess it. Is there an estimate of variation within each group of data? Is the variance similar between the groups that are being statistically compared?	Yes	Figure legends and Materials and Methods

Sample definition and in-laboratory replication	Information included in the manuscript?	In which section is the information available? (Reagents and Tools Table, Materials and Methods, Figures, Data Availability Section)
In the figure legends: state number of times the experiment was **replicated** in laboratory.	Not Applicable	
In the figure legends: define whether data describe **technical or biological replicates**.	Not Applicable	

Ethics

Ethics	Information included in the manuscript?	In which section is the information available? (Reagents and Tools Table, Materials and Methods, Figures, Data Availability Section)
Studies involving **human participants**: State details of **authority granting ethics approval** (IRB or equivalent committee(s), provide reference number for approval.	Not Applicable	
Studies involving **human participants**: Include a statement confirming that **informed consent** was obtained from all subjects and that the experiments conformed to the principles set out in the WMA Declaration of Helsinki and the Department of Health and Human Services Belmont Report.	Not Applicable	
Studies involving **human participants:** For publication of **patient photos**, include a statement confirming that consent to publish was obtained.	Not Applicable	
Studies involving experimental **animals**: State details of **authority granting ethics approva**l (IRB or equivalent committee(s), provide reference number for approval. Include a statement of compliance with ethical regulations.	Not Applicable	
Studies involving **specimen and field samples:** State if relevant **permits** obtained, provide details of authority approving study; if none were required, explain why.	Not Applicable	

Dual Use Research of Concern (DURC)	Information included in the manuscript?	In which section is the information available? (Reagents and Tools Table, Materials and Methods, Figures, Data Availability Section)
Could your study fall under dual use research restrictions? Please check biosecurity documents and list of **select agents and toxins** (CDC): https://www.selectagents.gov/sat/list.htm	Not Applicable	
If you used a select agent, is the security level of the lab appropriate and reported in the manuscript?	Not Applicable	
If a study is subject to dual use research of concern regulations, is the name of the **authority granting approval and reference number** for the regulatory approval provided in the manuscript?	Not Applicable	

Reporting

The MDAR framework recommends adoption of discipline-specific guidelines, established and endorsed through community initiatives. Journals have their own policy about requiring specific guidelines and recommendations to complement MDAR.

Adherence to community standards	Information included in the manuscript?	In which section is the information available? (Reagents and Tools Table, Materials and Methods, Figures, Data Availability Section)
State if relevant guidelines or checklists (e.g., **ICMJE, MIBBI, ARRIVE, PRISMA**) have been followed or provided.	Not Applicable	
For **tumor marker prognostic studies,** we recommend that you follow the **REMARK** reporting guidelines (see link list at top right). See author guidelines, under 'Reporting Guidelines'. Please confirm you have followed these guidelines.	Not Applicable	
For **phase II and III randomized controlled trials**, please refer to the **CONSORT** flow diagram (see link list at top right) and submit the CONSORT checklist (see link list at top right) with your submission. See author guidelines, under 'Reporting Guidelines'. Please confirm you have submitted this list.	Not Applicable	

Data Availability

Data availability	Information included in the manuscript?	In which section is the information available? (Reagents and Tools Table, Materials and Methods, Figures, Data Availability Section)
Have **primary datasets** been deposited according to the journal's guidelines (see 'Data Deposition' section) and the respective accession numbers provided in the Data Availability Section?	Yes	Data availability section
Were **human clinical and genomic datasets** deposited in a public access-controlled repository in accordance to ethical obligations to the patients and to the applicable consent agreement?	Not Applicable	
Are **computational models** that are central and integral to a study available without restrictions in a machine-readable form? Were the relevant accession numbers or links provided?	Yes	Data availability section
If publicly available data were reused, provide the respective **data citations in the reference list**.	Yes	Reference section
